# Mesoscale carbon fiber lattices with foam-like weight and bulk strength

**DOI:** 10.1038/s41467-026-72105-4

**Published:** 2026-04-21

**Authors:** Jun Young Choi, Sung-Hoon Ahn

**Affiliations:** 1 Department of Mechanical Engineering, Seoul National University, Seoul, South Korea; 2 Institute of Advanced Machines and Design, Seoul National University, Seoul, South Korea

**Keywords:** Composites, Mechanical engineering

## Abstract

Carbon-fiber-reinforced polymers (CFRPs) are essential for lightweight transport and energy systems, but most current forms—particulate, short-fiber, or laminated— break the continuity of reinforcing fibers, interrupting load transfer and limiting strength, safety, and design freedom. Architected lattice materials offer a route to higher strength-to-weight ratios, yet prior CFRP lattices are largely confined to the microscale or rely on joints and segmented fibers that compromise load transfer. Here we demonstrate fully continuous three-dimensional CFRP lattices fabricated at the mesoscale using a 3D node winding process guided by algorithmic design. By systematically controlling fiber continuity at the lattice unit-cell level, these structures achieve specific strengths of up to 782 MPa·cm³·g⁻¹ at foam-like densities, representing a considerable achievement in mesoscale CFRP lattice architectures. Unlike conventional CFRPs, the lattices fail progressively through pseudo-ductile, damage-tolerant mechanisms with partial height recovery under compression. System-level demonstrations, including a robotic drone with substantially reduced frame mass and extended endurance, confirm scalability and practical relevance. This work establishes continuity-engineered CFRP lattices as a promising class of lightweight architected materials for next-generation structural systems.

## Abstract (web summary)

Lightweight materials typically sacrifice strength for low density. Here, authors report mesoscale carbon-fiber lattices with continuous three-dimensional fiber pathways, achieving foam-like weight while approaching the strength of bulk composites.

## Introduction

CFRPs are central to lightweight structures in aerospace, automotive, and energy applications, valued for their high strength-to-weight ratios and durability^1,2^. Most structural CFRPs are still manufactured as laminates, where fibers are discretized into stacked plies. Although this approach enables high in-plane properties, interrupted through-thickness load paths create weak interlaminar regions that promote delamination and brittle failure, particularly under compression^3,4^. Recent advances in additive manufacturing—including curved-layer deposition, in-nozzle impregnation, and high–fiber–volume–fraction 3D printing—have expanded geometric freedom and enabled printed CFRPs with fiber contents approaching those of conventional composites^5–7^. However, these approaches remain fundamentally layer-wise, resulting in segmented fiber trajectories, interlayer interfaces, and limited three-dimensional load transfer. As a result, the exceptional intrinsic properties of carbon fibers are only partially exploited in current composite architectures.

Architected lattice materials offer a promising route to overcome these limitations by redistributing loads through open-cell geometries^8,9^. At the micro- and nanoscale, lithographic techniques have produced carbon nanolattices with extraordinary mechanical efficiency^10–12^, but these methods remain slow, costly, and limited to small-scale specimens. At the mesoscale, approaches based on mechanical fasteners, snap-fit connections, or additive manufacturing have enabled larger CFRP lattices^13–17^, yet suffer from discontinuous fiber paths, stress concentrations at joints, or weak interlayer adhesion. Reported strengths, therefore, remain far below the intrinsic capability of carbon fibers, leaving a significant performance gap between laboratory demonstrations and real-world structural applications^18^.

Coreless filament winding provides an alternative paradigm by enabling continuous fibers to be steered through three-dimensional space without mandrels and has been demonstrated in architectural-scale systems and hybrid structures^19–23^. Recent advances, including additive fiber tethering (AFT), have further expanded the design space for continuous-fiber mesoscale architectures by enabling trajectory-defined fiber placement for complex component geometries^24^. While these approaches successfully demonstrate uninterrupted fiber paths, they are primarily formulated around geometry-driven or component-level fabrication strategies. Building on this paradigm, we show that mesoscale CFRP lattices can approach near-intrinsic carbon-fiber efficiency when fiber continuity is systematically engineered and quantified at the unit-cell and node level. To achieve this, we introduced 3D node winding (3NW), a lattice-oriented variant of coreless filament winding in which a single continuous fiber is wound through lattice vertices (nodes) to form fully three-dimensional structures, treating the unit cell as the fundamental design primitive. By preserving uninterrupted fiber trajectories through load-bearing three-dimensional nodes while explicitly managing nodal congestion and lattice-level load redistribution, 3NW enables continuity-controlled mechanical scaling across foam-like relative densities.

Using 3NW, we fabricated simple cubic (SC) and face-centered cubic (FCC) CFRP lattices that achieve foam-like relative densities while exhibiting specific compressive strengths of up to 782 MPa·cm³·g⁻¹, approaching iso-specific-strength limits of bulk CFRP. This behavior is rationalized through a continuity-based scaling framework that extends classical Gibson–Ashby models to account for uninterrupted load transfer across lattice nodes. These lattices fail through progressive, damage-tolerant mechanisms with partial height recovery rather than brittle collapse. System-level demonstrations in a robotic drone further confirm that maximizing fiber continuity at the lattice scale directly translates into lighter, safer, and more energy-efficient structures.

## Results

### Manufacturing strategy for mesoscale CFRP lattices

Achieving continuous fiber reinforcement in three dimensions requires a fabrication strategy that avoids the discontinuities^25^ inherent in existing mesoscale CFRP lattices. We developed a modular approach, termed 3NW, that combines 3D-printed supports with continuous fiber placement to preserve uninterrupted paths across lattice nodes.

**Fig. 1:**
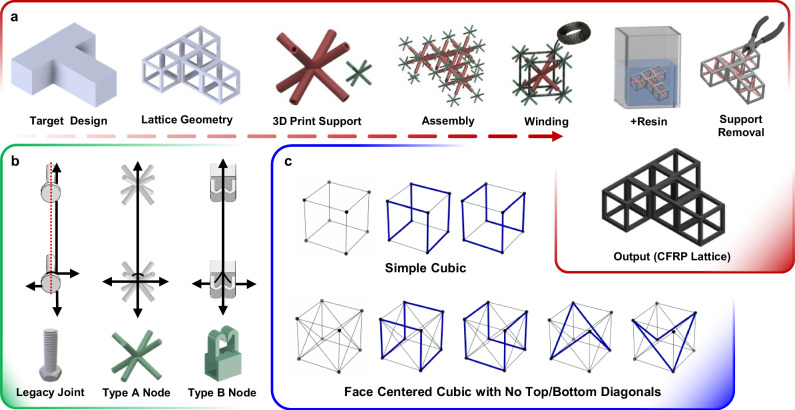
Fabrication and design logic of continuous CFRP lattices. **a** Target structures are decomposed into modular cores and nodes, 3D-printed as sacrificial supports, wound in a single roving pass, resin-impregnated, and released to yield joint-free lattices. **b** Node designs. Legacy bolt-type guides misalign fibers, while Type-A isotropic BCC nodes interlock with hollow cores and Type-B clip-lock nodes enable 2D–2.5D assemblies. **c** Modular winding sequences for SC and FCC lattices without top–bottom diagonals, with vertical struts looped twice to preserve fiber continuity.

The process begins with the selection of a target lattice geometry and the design of modular cores and nodes that define discrete fiber anchoring locations (Fig. 1a). These supports are fabricated by fused-filament 3D printing and assembled into a scaffold that maintains geometric alignment during winding. Carbon-fiber roving is then placed continuously along predefined paths under controlled tension to ensure stable alignment and prevent slack formation. All winding parameters—including tension, winding speed, path angle, and node engagement—were quantitatively defined and monitored (Supplementary Method S1). Following manual winding, the lattices are resin-impregnated and cured to yield fully consolidated composite structures.

Two complementary support systems were developed (Fig. 1b). Legacy bolt-type joints^26^ forced fibers to wrap in circular paths, often inducing misalignment and local stress concentrations during three-dimensional winding. In contrast, Type A nodes employ a body-centered cubic (BCC) derived geometry that anchors fibers along up to seven directions while maintaining straight paths through the node, enabling true three-dimensional connectivity. Type B nodes use clip-lock features that constrain fibers in one or two directions, simplifying handling and accelerating assembly. Together, these node designs improve fiber alignment and reduce nodal defects relative to legacy winding aids.

Candidate lattice topologies are summarized in Supplementary Table 1. SC and FCC were selected as benchmarks because they balance connectivity with manageable node congestion, whereas BCC lattices concentrate overlaps at a central node, and triangular or hexagonal prisms suffer from stacking and geometric incompatibility. All unit-cell–level experiments, therefore, employ SC and FCC cells fabricated by 3NW. Single unit cells were fabricated through sequences shown in Fig.1c. A compact classification of tested unit cells is provided in Supplementary Table 2.

Fabrication reproducibility was assessed by producing ten nominally identical SC lattices under identical winding and curing conditions (Supplementary Fig. 1; Supplementary Method S2). Peak compressive force varied by ±11.6% and strength-to-weight ratio by ±8.3%, with consistent stiffness, failure modes, and no statistical outliers. Void content analysis of polished cross-sections yielded a low average porosity of 2.75 ± 1.12 vol%, comparable to filament-wound CFRP (Supplementary Method S3; Supplementary Fig. 2). Together, these results indicate that continuity-governed lattice performance is robust to the fabrication variability inherent to manual winding.

To explore scalability beyond single cells, a shortest-path algorithm was used to minimize redundant traversals during winding (Supplementary Fig. 3; Supplementary Method S4). For multi-cell assemblies and system-level structures, derived variants incorporating diagonal-only (DC) and hybrid SC–DC connectivity were additionally explored within the same nodal and continuity framework. Preliminary multi-cube assemblies confirmed the feasibility of modular integration (Supplementary Fig. 4; Supplementary Method S5), although small FCC cells exhibited congestion that limited fabrication quality. These results highlight the importance of algorithmic optimization for scaling to larger assemblies. Overall, 3NW maintains fiber continuity across all dimensions, unlike fastener-based or additive methods that introduce joints or segmented paths, thereby recovering the intrinsic compressive efficiency of carbon fibers while remaining geometrically versatile and scalable.

### Mechanical scaling and performance

The compressive performance of architected lattices is conventionally interpreted using Gibson–Ashby^27^ relations, where normalized strength scales with relative density, $\sigma \propto \bar{\rho }^{n}$, with $n=1$ for stretch-dominated members and $n=2$ for elastic buckling of slender struts. Here, relative density ($\bar{\rho }$) is defined as the mass of the cured, load-bearing CFRP lattice normalized by the equivalent volume of fully dense CFRP occupying the same external bounding volume. Relative density was calculated from Eq. 2 using measured mass and geometry. While this framework defines theoretical envelopes, it assumes perfect continuity across nodes. In practice, mesoscale CFRP lattices fabricated by printing, fasteners, or resin-rich joints lose continuity and therefore fall well below these limits.

To capture this effect, we introduce a continuity factor η (Eq. 4), which rescales the Gibson–Ashby law to reflect architecture- and process-dependent load transfer efficiency. Conceptually, $\eta \to 1$ for uninterrupted wound paths, whereas laminates, short-fiber, and particulate composites occupy progressively lower bands. This transforms single scaling curves into continuity-bounded performance regimes (Supplementary Fig. 5a–c, Supplementary Method S6).

Topology selection reflects this logic. SC lattices minimize overlap congestion (three overlaps per corner), stack cleanly, and favor high η. FCC lattices add diagonals that stabilize nodes and shift toward stretch dominance but incur more overlaps (six per edge) and modestly reduced η. BCC lattices concentrate eight overlaps at a central node, leading to severe congestion and were therefore excluded.

**Fig. 2:**
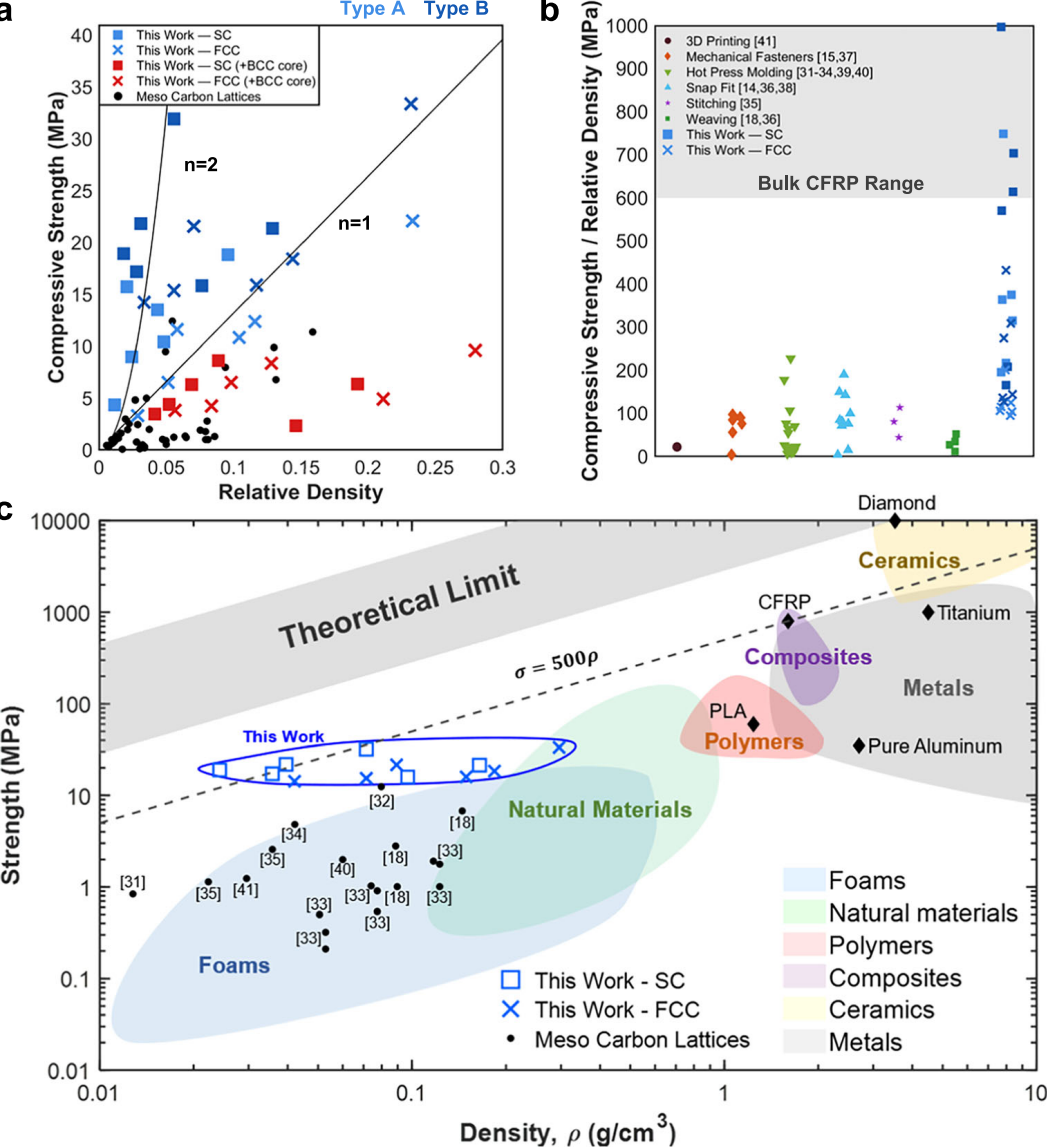
Compressive performance of continuous CFRP lattices. **a** Stress–density scaling shows SC approaching *n* = 2 and FCC *n* = 1 trends, both surpassing prior meso carbon lattices^14,15,18,37–47^. Each data point corresponds to the mean value obtained from at least three independently fabricated specimens per configuration. **b** Normalized strength-to-density ratios approach bulk CFRP. **c** Ashby chart positions continuous lattices near the theoretical efficiency limit, entering an unoccupied regime for ultra-light structures.

Figure 2a–c summarizes compressive performance. Continuous SC and FCC lattices populate a previously sparse regime of foam-like densities ($\bar{\rho }\approx 0.02-0.10$) combined with high specific strength. SC datasets align with a slope near $n\approx 2$ but at substantially higher prefactor. FCC data trend toward $n\approx 1$ with greater scatter, with greater scatter due to diagonal load sharing and overlap penalties. Normalized plots (Fig. 2b) show SC specimens at $\bar{\rho }\approx 0.056$ reaching $\sigma \approx 32$ MPa, over an order of magnitude above reported mesoscale CFRPs at similar densities. Several points approach or exceed iso-specific-strength lines of bulk CFRP^28–30^, demonstrating that continuity enables mesostructures to rival the parent composite.

The material system also influenced performance. Type B lattices, built with higher-modulus fiber and lighter resin, consistently outperformed Type A baselines. Nevertheless, topology remained the dominant lever: SC generally gave the highest specific strengths because its lower node congestion preserved fixity and higher η; FCC lattices could sustain larger absolute loads at a given size but were more sensitive to misalignment and resin pooling. Representative force–displacement curves are shown in Supplementary Fig. 6a–g.

Placing the data on an Ashby chart^31^ (Fig. 2c) highlights the broader significance. Continuous CFRP lattices extend into a high-specific-strength window at mesoscales where microscale lithographic lattices rarely scale in absolute size, while remaining far more manufacturable. By maximizing continuity, lattices move upward within the Gibson–Ashby bands, reducing stress concentrations at nodes and converting brittle joint failure into progressive column collapse.

Finally, measured loads were compared with Euler predictions using member areas from image analysis and imperfection factors tuned by loop count and gauge length. Long slender members followed Euler scaling, whereas shorter cells deviated due to local crushing and waviness. These comparisons, detailed in Supplementary Methods S7–S8, confirm that continuity and fabrication quality—rather than topology alone—govern performance. Supporting data include compressive responses of single columns and SC lattices (Supplementary Fig. 7a–f), cross-sectional properties (Supplementary Table 4), column strengths versus Euler predictions (Supplementary Table 5), and comparisons of experimental results for SC and FCC lattices with imperfection-adjusted Euler theory (Supplementary Table 6). Upward departures relative to the mesoscale state of the art are therefore attributed to recovered continuity, not boundary-condition artifacts.

### Failure modes and damage tolerance

**Fig. 3:**
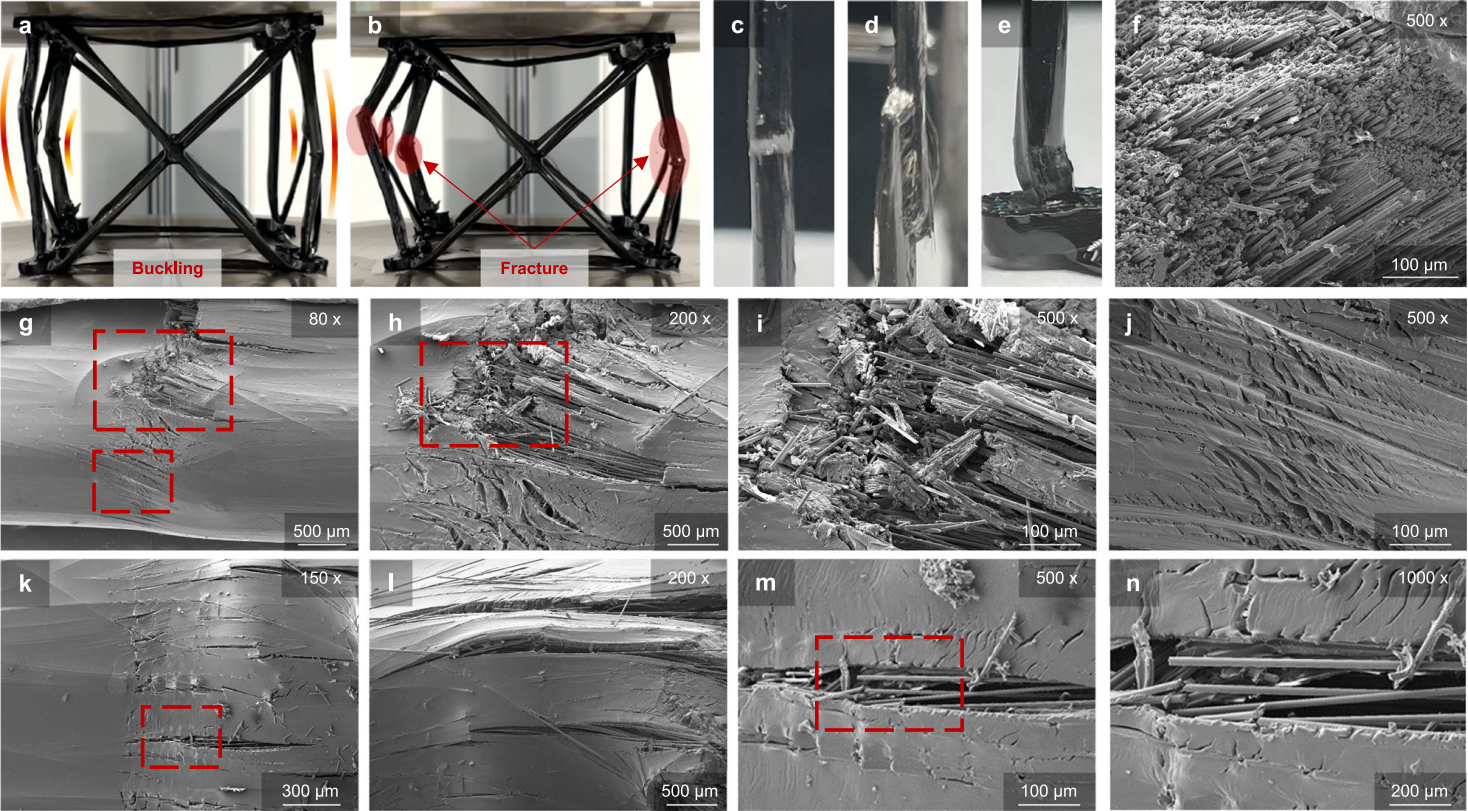
Failure behavior of CFRP lattices. **a, b** Compression of a 50 mm FCC lattice showing buckling followed by snap fracture. **c–e** Optical images of buckle failure, delamination, and kink-crushing. **f** SEM cross-section of failed columns. **g–j** SEM views of buckle failure at increasing magnifications, showing fiber fracture and resin cracking. **k–n** SEM views of kink-crushing, from low to high magnification, highlighting progressive kink-band collapse. SEM observations were obtained from three independently tested lattice specimens (*n* = 3) with similar failure features; the images shown are representative.

The collapse of continuous CFRP lattices departs from the brittle, catastrophic fracture of conventional laminates. While isolated slender tows followed Euler predictions at high slenderness (Supplementary Video 1), whole lattices failed through mixed mechanisms shaped by fiber continuity, nodal fixity, and geometric imperfections (Supplementary Video 2). Cross-sectional imaging confirmed that each column contained thousands of initially well-aligned fibers (Fig. 3f), underscoring the structural efficiency of the wound architecture and the role of fiber alignment in shaping failure.

Optical views captured the macroscopic manifestations of these modes (Fig. 3c–e). Local buckling produced visible surface bulges, resin whitening, and delamination, particularly in thicker verticals. These images highlight how deformation extended over multiple members without immediate fiber rupture, although resolution was insufficient to capture fine details.

At higher resolution, three recurring categories emerged (Fig. 3a–b, g–n). In 50-mm lattices, global bowing drove buckling–snap, where resin first cracked along the fiber axis before sudden transverse breaks severed columns (Fig. 3a, b, g–j). Near nodes, deformation localized into kink-crushing. Between the fracture plane and the node, severe longitudinal matrix cracking developed, with fibers remaining continuous. At the fracture region itself, ripple-like kink bands formed, characterized by matrix shear cracking and fiber misalignment (Fig. 3k–n). Finally, oblique cracking with delamination occurred in thicker verticals (Fig. 3d), where diagonal separation and resin whitening relieved stress without full fiber severance. Supplementary Video 3 provides additional SEM zoom-in/out views of buckling and kinking fracture.

The load redistribution seen in Supplementary Video 2 confirmed this hierarchy: lattices often maintained or even increased load after the first column fractured, collapsing only once multiple verticals failed. Loop number amplified severity—two-loop specimens bore higher loads but transitioned more abruptly once instability initiated—yet the governing mode was set by geometry.

A notable feature was partial dimensional recovery after unloading. Because most cracks aligned with fiber direction rather than cutting across it, many fibers remained intact, allowing misaligned columns to elastically straighten. Redundant members delayed catastrophic collapse, producing a pseudo-ductile response absent from conventional laminates.

This damage tolerance was further validated under repeated loading. Displacement-controlled cyclic compression tests performed to 50% of the maximum monotonic deformation demonstrated stable hysteresis behavior without catastrophic failure. Buckle-dominated lattices exhibited gradual pseudo-hardening over successive cycles with negligible permanent height reduction, consistent with stabilization of buckled members into repeatable load paths. In contrast, kink-crush-dominated lattices showed pronounced initial stiffening followed by controlled softening associated with progressive densification, while retaining substantially higher stiffness and load-bearing capacity (Supplementary Fig. 8; Supplementary Method S9).

Complementary force-controlled cyclic tests over 50 cycles further confirmed dimensional stability in both architectures. Under fixed load amplitudes, neither lattice exhibited progressive collapse or loss of structural integrity, indicating that both buckling- and kink-crushing-dominated deformation modes are accommodated through stable, repeatable mechanisms rather than brittle fracture.

In sum, continuous-fiber CFRP lattices fail progressively rather than catastrophically. By preserving fiber continuity across nodes and limiting complete fiber rupture, they combine high strength with pseudo-ductile, partially reversible failure behavior under both monotonic and cyclic loading, enabling safer and more damage-tolerant performance than conventional laminated composites.

### Lightweight drone demonstrator

The quadcopter airframe provides a clear test case for 3NW, since it dictates overall weight, load-carrying strength, and stability. A practical frame must remain light to maximize endurance yet strong to resist collapse and stiff to suppress vibrations. To assess these requirements, we first benchmarked lattice beams for stiffness-to-mass efficiency before applying them to drone-scale airframes.

**Fig. 4:**
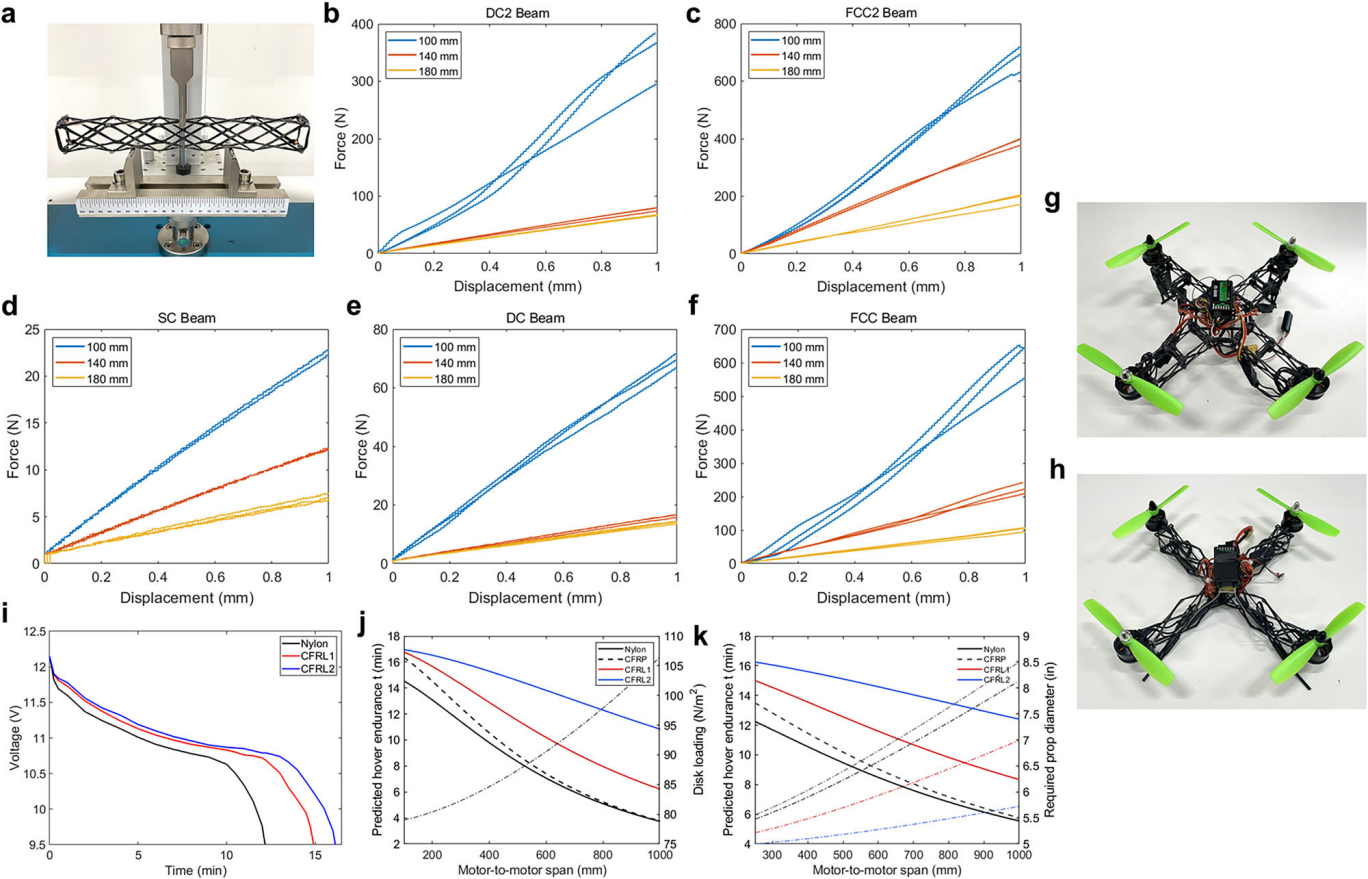
Structural performance and drone demonstration. **a** Three-point bending setup. **b–f** Force–displacement curves reveal topology-dependent stiffness. **g, h** Drone frames from FCC hybrid (60 g) and optimized DC–SC hybrid (21 g). **i** Hover tests show endurance gains of CFRP-lattice drones over nylon. **j** Endurance scaling with fixed 5-inch propellers. **k** Constant-disk-loading case with resized propellers, confirming sustained lattice advantage.

Three 250-mm beam types were fabricated: SC, DC, and FCC. SC beams were the weakest in bending, FCC beams achieved very high stiffness but required excessive fiber and were difficult to demold, while DC beams provided the best balance of stiffness and manufacturability (Supplementary Table 8). Optimization under an 8 g cap identified a DC beam (22 × 30 mm, five cells, loop-1 winding) as the top-ranked design (Fig. 4a–f; Supplementary Table 9). This guided drone integration, where beams were crossed into X-shaped frames.

Three airframes were tested with identical propulsion systems (Supplementary Video 4). The nylon baseline used a 100 g injection-molded frame, compared to 60 g for CFRL1, a simplified FCC lattice, and 21 g for CFRL2, a DC–SC hybrid derived from the optimized design (Fig. 4g, h). Hover tests (Fig. 4i) showed endurance of 12.25 min for nylon, 15.0 min for CFRL1 ( + 22 %), and 16.25 min for CFRL2 ( + 33 %). Average power consumption dropped from 107.7 W (nylon) to 88.0 W (CFRL1) and 81.2 W (CFRL2), confirming that lattice frames substantially reduced energy demand. A full comparison is provided in Supplementary Table 10.

Scaling analysis^32^ ($P\propto {m}^{3/2}$) predicted power reductions of only 13.7 % and 23.9 % for CFRL1 and CFRL2, whereas measured decreases were 18.3 % and 24.6 %. The discrepancy reflects the disproportionate role of the frame: it accounted for 20 % of the total mass in nylon but only 5 % in CFRL2. Beyond weight savings, lattices provided secondary gains from higher stiffness, which suppressed vibrational losses, and reduced drag from the open geometry.

Scalability was assessed by modeling endurance versus span L (Fig. 4j, k; Supplementary Methods S10). With fixed 5-inch rotors (Fig. 4j), endurance decreased with size, but CFRL2 consistently maintained the highest margin, while solid CFRP lay between nylon and the lattice variants. In the constant-disk-loading case (Fig. 4k), propeller diameter was resized to maintain *DL*_*250*_. CFRL2 required only a modest increase, from ~5.0 in at 250 mm to ~5.7 in at 1000 mm, whereas nylon and CFRP frames demanded much larger props (up to ~8–8.5 in). This reflects the lower mass of the lattice frames, with CFRL2 again sustaining the greatest endurance across the span range. These results confirm that lattice efficiency gains extend from meso-scale drones toward larger platforms constrained by stiffness.

Additional prototypes demonstrated versatility. A robotic arm link (Supplementary Fig. 9; Supplementary Methods S11) lifted 17.49 N versus 15.94 N for a PLA baseline while reducing mass by 53.7 %. Shortest-path winding applied to 2D and triangular-prism cells (Supplementary Fig. 10) highlighted modular adaptability. A miniature aircraft prototype (Supplementary Fig. 11; Supplementary Method S12) with a 500-mm wingspan and lattice-derived Clark-Y airfoil confirmed the aeronautical potential of 3NW.

## Discussion

This study demonstrates that continuous CFRP lattices can be fabricated at the mesoscale using 3D node winding, overcoming the discontinuities that limit most existing architectures. Within the Gibson–Ashby model, continuity emerges as the decisive variable that shifts performance toward the efficiency band of bulk composites, while discontinuous systems remain confined to lower regimes.

Failure also differs from conventional laminates. Instead of brittle delamination, lattices deform through kink bands, bowing, and oblique cracking, leaving nodes intact and allowing partial recovery of height after unloading. This pseudo-ductile behavior suggests a safer failure pathway, with damage signaled through visible deformation rather than sudden collapse.

System-level validation with drone airframes confirms that continuity at the mesostructural scale translates directly into lighter, more efficient vehicles. More broadly, robotic winding and automated path planning could enable larger, more consistent structures, extending this approach beyond laboratory specimens toward practical applications in aerospace and robotics.

## Methods

### CFRP lattice fabrication

CFRP lattices were manufactured using a 3D node winding (3NW) process in which modular cores and nodes, produced by fused filament 3D printing, acted as sacrificial supports. Carbon fiber roving was wound continuously around the assembled scaffold according to modular path orders, ensuring uninterrupted reinforcement across edges and diagonals. After winding, lattices were resin-impregnated and cured to form consolidated composite architectures.

Two material systems were employed. Type A lattices combined TANSOME H2550-24K carbon fiber roving (tensile strength ~5.5 GPa, modulus 250 GPa, density 1.80 g/cm³) with a commercial bisphenol-A epoxy (ENPOXI Sports S, viscosity ~220 cps). Type B lattices used higher-performance TANSOME H3060-24K roving (tensile strength ~6.1 GPa, modulus 290 GPa, density 1.78 g/cm³) together with a bisphenol-F epoxy (Kukdo KFR-5121 with KH-818B hardener). During winding, ~3–5 N of tension was maintained to ensure alignment and pre-strain, and resin infiltration was achieved by immersion and gravity drainage.

### Additive support structures

The additive support structures consisted of modular cores and nodes designed to withstand winding tension while fixing fiber paths with high positional accuracy. Supports were fabricated using a Bambu X1 Carbon printer (Bambu Lab, China) with PLA filament (density 1.24 g cm⁻³, 15% infill, 0.4 mm nozzle, 0.2 mm layer height). A 35° support threshold was applied to stabilize overhangs.

Type A supports were based on hollow BCC cores connected through smaller solid BCC nodes. Each node provided up to seven anchoring directions, guiding fibers along both edges and diagonals. This configuration enabled true three-dimensional connectivity, ensured continuous reinforcement through SC and FCC unit cells, and reduced the stress concentrations that arise when fibers are wrapped around bolts or pins.

Type B supports used simple-cubic cores reinforced with 3 × 3 mm ribs that projected from each face. These SC-style ribbed cores could be printed as single integrated blocks or as modular units. They were paired with clip-lock nodes shaped for one-way fiber entry, anchoring fibers in only one or two directions. Although less isotropic than Type A, this approach was easier to print, handle, and demold, making it well-suited for quasi-2D or 2.5D assemblies and for rapid prototyping.

Together, Type A and Type B provided complementary capabilities: Type A maximized fiber continuity and design freedom for complex 3D lattices, while Type B enabled faster, more practical testing of simplified architectures.

### Shortest path winding

A shortest-path strategy was implemented to generate continuous fiber toolpaths while minimizing redundant traversals. Each lattice was represented as a weighted graph$\,G=\left(V,E\right)$, with vertices as nodes and edges as struts. Candidate edge sequences were reconstructed into continuous traversals by inserting shortest connectors, and the total path length

1 $${L}_{{path}}={\sum }_{\left(i,j\right)\in {{\wp }}}{w}_{{ij}}$$

was minimized by a genetic algorithm (50 individuals, 200 generations). The optimized path was then segmented by unit-cell membership to produce fabrication-ready winding modules.

Alternative formulations for continuous-fiber path planning—including turning-angle–optimized paths^33^ and learning-based planners^34^, have been reported primarily in the context of additive manufacturing and layer- or surface-constrained deposition. Here, we address fully three-dimensional, node-constrained winding graphs that require uninterrupted fiber continuity, deliberate edge revisits, and compatibility with manual or semi-manual winding. Under these constraints, ensuring global path feasibility and continuity preservation is prioritized over local geometric optimality; therefore, a genetic algorithm was selected for its robustness and flexibility across diverse lattice topologies.

A representative workflow is shown in Supplementary Fig. 3, extended algorithm details are in Supplementary Method 4, and the full code is provided as Supplementary Code 1.

### Relative volume and density

Relative density ($\bar{\rho }$) was defined as the mass of the cured, load-bearing CFRP lattice normalized by the mass of a fully dense CFRP solid occupying the same external bounding volume:

2 $$\bar{\rho }\,=\,\frac{{m}_{{CFRP}}}{{\rho }_{{CFRP}}{V}_{{bulk}}}$$

where ${m}_{{CFRP}}$ is the measured mass of the cured lattice after complete removal of sacrificial supports, ${\rho }_{{CFRP}}$ is the density of the fully cured CFRP material, and ${V}_{{bulk}}$ is the external bounding volume defined by the lattice envelope.

For homogeneous CFRP lattices, this definition is equivalent to the solid volume fraction. For hybrid structures in which non-load-bearing PLA components (e.g., BCC support cores) are retained, a relative volume fraction is reported instead, defined as:

3 $$\bar{V}=\frac{{V}_{{CFRP}}+{V}_{{polymer}}}{{V}_{{bulk}}}$$

This metric is used solely to describe geometric occupancy and mass distribution; all mechanical scaling, Gibson–Ashby analysis, and Ashby-chart comparisons are based exclusively on CFRP relative density.

In Fig. 2c, the absolute material density (lattice density) plotted on the Ashby chart is obtained as ${\rho=\bar{\rho }\rho }_{{CFRP}}$, ensuring consistent comparison with literature data for bulk materials and architected composites.

### Gibson Ashby scaling and continuity factor

The mechanical performance of architected lattices is conventionally interpreted using the Gibson–Ashby framework, where normalized strength scales with relative density,

4 $${\sigma }_{{lat}}\propto {\bar{\rho }}^{n}$$

with $n=1$ for stretch-dominated and $n=2$ for buckling-dominated struts. SC lattices with long vertical members tend toward the buckling regime, while FCC lattices, stabilized by diagonals, shift toward stretch-dominated behavior.

Classical scaling assumes perfect continuity across nodes. In practice, fabrication routes introduce discontinuities that reduce load transfer and depress strengths below these bounds. To capture this, we introduce a continuity factor η as a scalar efficiency that rescales the Gibson–Ashby law:

5 $${\sigma }_{{lat}}=C{E}_{c}^{\left(n\right)}{\eta }_{{{\rm{eff}}}}\left(n\right){\bar{\rho }}^{n},\,n=\left\{1{{\rm{stretch}}},\,2{{\rm{buckling}}}\right\}$$

where C is a geometric constant, ${E}_{c}^{(n)}$ is the effective modulus projected along the loading direction, and ${\eta }_{{{\rm{eff}}}}(n)$ reflects directional continuity.

Continuity estimators for wound lattices, laminates, short-fiber, and particulate composites are provided in Supplementary Method S6 (Eq. S4–S10), with the estimated continuity for each case in Supplementary Table 3. These establish η→1 for uninterrupted winding, reduced values for congested topologies (e.g., BCC), and much lower bands for discontinuous systems.

### Uniaxial compression testing

Due to the absence of a dedicated compressive testing standard for composite or hybrid composite lattice architectures, ASTM D1621—commonly applied to cellular and polymer foams—was adopted as a reference methodology. This standard has been widely applied to evaluate the compressive response of polymer and composite lattices, making it a suitable benchmark for the present study^35,36^. Lattice specimens were prepared with dimensions guided by ASTM recommendations, with standard sample sizes of 25, 37, and 50 mm. All compressive tests were performed using a universal testing machine under displacement control at a constant crosshead speed of 3 mm/min. The primary objective was to evaluate the peak compressive strength and progressive failure modes of composite lattice geometries under uniaxial loading.

Since the cross-sectional area of lattices varies along their height, the effective area was approximated by dividing the total volume of each constituent material (PLA and CFRP) by the specimen height. The volumes were determined from the measured mass and corresponding densities (${{{\rm{\rho }}}}_{{{\rm{PLA}}}}=1.24\frac{g}{c{m}^{3}}$, ${\rho }_{{CFRP}}\approx 1.6\frac{g}{c{m}^{3}}$). The total cross-sectional area was then expressed as:

6 $${A}_{{tot}}= {A}_{{PLA}}+{A}_{{CFRP}}= \frac{{V}_{{PLA}}}{h},+\frac{{V}_{{CFRP}}}{h}$$

The compressive stress was calculated by normalizing the applied force with respect to this effective area:

7 $${\sigma }_{c}=\frac{F}{{A}_{{PLA}}+{A}_{{CFRP}}}$$

This method accounts for both polymeric supports and CFRP reinforcement, enabling a consistent stress evaluation across lattice geometries with varying material proportions and cross-sectional heterogeneity.

Unless otherwise stated, all mechanical tests were performed on at least three independently fabricated specimens per configuration.

### Microscopic imaging

Fracture surfaces and deformation morphologies of CFRP lattices were examined using a field-emission scanning electron microscope (FE-SEM, SIGMA, Carl Zeiss, UK; installed 2014). The system operates with an accelerating voltage range of 0.1–30 kV and a resolution of 1.0 nm at 15 kV. Imaging was conducted in high-vacuum mode with typical beam energies of 12–15 kV. An Everhart–Thornley secondary electron detector was used to resolve surface features, while an in-lens detector captured high-resolution microstructural details. Representative magnifications ranged from 80× to 1000× (Fig. 3g–n), allowing observation of kink bands, resin cracks, and fiber fractures at multiple length scales.

### Stiffness measurement and beam optimization

To translate unit-cell behavior into system-level structures, we characterized the bending stiffness of lattice beams and optimized their geometry for use in a 250-mm-class quadrotor drone. Each beam was fabricated by serially connecting five unit cells (50 mm each) to form a total length of 250 mm. Two interchangeable cross-sections (22 × 30 mm and 30 × 22 mm) were considered to probe aspect-ratio effects. Beams were manufactured using Type B support structures in three lattice types: SC with one loop per edge (SC-1), diagonal-only (DC-1 and DC-2), and FCC with one or two loops (FCC-1 and FCC-2).

Three-point bending tests were performed at a displacement rate of 2 mm min⁻¹, with the loading point applied at the central node. Spans of 100, 140, and 180 mm were tested to separate bending and shear contributions. Apparent stiffness was extracted as the slope of the 0–1 mm region of the force–displacement curve. SC beams consistently gave the lowest stiffness, FCC beams reached the highest values but required excessive fiber and posed demolding challenges, while DC beams offered the most practical balance of stiffness and manufacturability.

The apparent stiffness $S$ at a given span was defined as

8 $$k\left(S\right)=\frac{\Delta F}{\Delta {{\rm{\delta }}}}\,\left[{\mbox{N}}/{\mbox{mm}}\right]$$

and modeled by Timoshenko beam theory as

9 $$\frac{1}{k\left(S\right)}=\frac{{S}^{3}}{48{EI}}+\frac{S}{4{{\rm{\kappa }}}{GA}},\,\kappa=\frac{5}{6}$$

where ${EI}$ is the effective flexural rigidity, ${GA}$ is the effective shear rigidity. By fitting this relation across three spans, effective stiffness constants were identified for each lattice. Swap tests between 22×30 mm and 30×22 mm sections, combined with loop-count comparisons, yielded empirical scaling laws:

10 $${EI}\propto {b}^{1}{h}^{4.7}{a}^{-1}{{\mbox{loops}}}^{2.1},\,{GA}\propto {b}^{1}{h}^{0.9}{a}^{-1}{{\mbox{loops}}}^{3.1}$$

where b is the beam width, ℎ the beam height, α the unit cell length, and “loops” the number of roving passes per cell. These relations confirmed that beam height is the dominant contributor to bending stiffness, while width plays a secondary role, and longer unit cells reduce rigidity.

Beam weight was predicted from the roving path length. For each unit cell, the total fiber length was computed geometrically, and the overall beam mass was given by

11 $${m}_{{\mbox{beam}}}=\left({\sum }_{i=1}^{5}{L}_{{\mbox{cell}},i}\right)\cdot \left({\mbox{loops}}\right)\cdot {\mbox{MPL}}$$

where ${L}_{{\mbox{cell}},i}$ is the path length of the *i*-th cell, and MPL is the calibrated mass per fiber length, equal to 0.003449 g/mm for loop−1 and 0.003562 g/mm for loop-2 configurations. This calibration was obtained from measured weights of beams of known length and loop count.

With stiffness and mass models established, the beam optimization was formulated as

12 $${\mbox{maximize}}\,k\,\,{\mbox{subject to}}:m\le 10{{\rm{g}}}$$

Design variables included beam width b, height h, and the five unit-cell lengths $({a}_{1},\cdots,{a}_{5})$ constrained such that $\sum {a}_{i}$ = 250 mm, exactly five cells were used, and the central cell length equalled the beam width ${a}_{3}=b$. Roughly 30,000 candidate beams were generated by random sampling within feasible bounds; overweight candidates were discarded. For each, stiffness was computed by piecewise Timoshenko integration over half-span:

13 $${{\rm{\delta }}}=\frac{{{\rm{P}}}}{2}{\int }_{0}^{{{\rm{S}}}/2}\frac{{{{\rm{x}}}}^{2}}{{{\rm{EI}}}\left({{\rm{x}}}\right)}{{\rm{dx}}}+\frac{{{\rm{P}}}}{2{{\rm{\kappa }}}}{\int }_{0}^{{{\rm{S}}}/2}\frac{{{\rm{dx}}}}{{{\rm{GA}}}\left({{\rm{x}}}\right)},\,{k}=\frac{1}{{{\rm{\delta }}}},\,{P}=1{{\rm{N}}}$$

he full code is in Supplementary Code 2, and he top five designs under the 8 g cap are listed in Supplementary Table 9.

### Drone endurance testing

The drone propulsion system used were MT2204 2300 kV motors, 5-inch propellers, 3S 2200 mAh LiPo. The electronics and propulsion set contributed a constant 404 g across all cases, so differences arose entirely from the frame. Hover endurance tests were conducted indoors under stabilized flight control using the LibrePilot GCS system (PID stabilization settings provided in Supplementary Code 3). Fully charged 3S LiPo packs (12.6 V, 4.2 V per cell) were used, and flight was maintained until the pack voltage reached the cutoff corresponding to 90 % depth of discharge ( ≈ 9.3–9.4 V).

Voltage–time traces were recorded in real time using a CVT01 telemetry sensor connected to the flight controller and plotted directly without additional correction. Capacity utilization u was extracted from the manufacturer’s discharge curve, and the average current and power were computed as

14 $$I=\frac{{uC}}{t/60},\,P=\frac{u{E}_{{nom}}}{t/60}$$

where *C* = 2.2 Ah, *t* = 10 min, and *E*_nom_ = 24.42 Wh, $t$ is flight time (min) and $u$ is the fraction of capacity consumed. Projected endurance to 90 % cutoff was calculated as

15 $$T=\frac{t{u}^{*}}{u},\,{u}^{*}=0.90$$

Endurance values for the three frame types were obtained using this procedure and are summarized in Supplementary Table 10.

### Reporting summary

Further information on research design is available in the Nature Portfolio Reporting Summary linked to this article.

## Supplementary information

Supplementary Information

Description of Additional Supplementary File

Supplementary Video 1

Supplementary Video 2

Supplementary Video 3

Supplementary Video 4

Supplementary Code 1

Supplementary Code 2

Supplementary Code 3

Reporting Summary

Transparent Peer Review file

## Source data

Source Data

## Data Availability

The data that support the findings of this study are available in the Main Text and Supplementary Information. Source data are provided with this paper.

## Code availability

The custom algorithms for winding path optimization, beam optimization, and lattice analysis are available in the Code Ocean repository at 10.24433/CO.0799073.v1. Supplementary Code 1–3 are also provided with this paper.
