# Supplementary material for: Mesoscale carbon fiber lattices with foam-like weight and bulk strength
Source: Nat Commun. 2026 Apr 21;17:3615. doi: 10.1038/s41467-026-72105-4 (PMC13100039; doi:10.1038/s41467-026-72105-4)
Supplement: Supplementary file 1 — Supplementary Information [file 41467_2026_72105_MOESM1_ESM.pdf]

Supplementary Information

Mesoscale Carbon Fiber Lattices with Foam-Like Weight and Bulk Strength

Jun Young Choi[a,b], Sung-Hoon Ahn[a,b]*

[a]Department of Mechanical Engineering, Seoul National University, Seoul, South Korea

[b]Institute of Advanced Machines and Design, Seoul National University, Seoul, South Korea

*Corresponding Author. *Email address:* ahnsh@snu.ac.kr

This PDF File Includes:

- Supplementary Methods 1-12
- Supplementary Figures 1-11
- Supplementary Table 1-10

Other Supplementary Information for this Manuscript Includes:

- Supplementary Movies 1-4
- Supplementary Codes 1-3
- Supplementary Data 1

Supplementary Methods

S1: Winding Parameters and Tensional Control

To ensure repeatability and mechanical consistency across all specimens, all key winding parameters—tension, winding speed, path angle, and node engagement—were quantitatively defined and monitored throughout fabrication.

Tensional Control

Winding tension was identified as the most critical parameter governing fiber alignment, geometric precision, and residual pre-strain. Carbon-fiber roving tension was maintained within a range of 3–5 N, determined through preliminary trials as the optimal window for stable placement without fraying, slack formation, or node slippage. Tensions below 3 N consistently produced local relaxation and uneven spans, whereas tensions above 5 N led to bundle flattening and occasional splitting at node–fiber interfaces.

Tension was monitored continuously using an in-line digital force gauge (FG-107-5K, AMITARRI, China) positioned between the roving spool and the support scaffold. During winding, the average tension remained within the target range, with transient fluctuations of ±0.3 N (≈6–10%), attributed primarily to frictional transitions at clip-lock nodes. These variations were well within acceptable limits for maintaining uniform pre-strain and geometric stability.

At the selected tension range, the elastic pre-strain imposed on the roving is estimated as

$$\varepsilon_r = \frac{T}{E_f A_f} \qquad \text{(S1)}$$

corresponding to $\varepsilon_r \approx (1-2) \times 10^{-5}$for the present fibers. This level of pre-strain is sufficient to suppress slack during impregnation and curing while remaining far below the fiber failure strain. Analytical estimates and empirical observation confirmed that gravitational sag between nodes was negligible (< few micrometers) for all tested spans.

Winding speed and path geometry

Winding speed was maintained at approximately 2 s per edge, regulated by metronome timing to ensure uniform pacing. This speed minimized jerking and tension loss while avoiding prolonged relaxation during winding. For the lattice geometries studied, this corresponded to ≈32 s per loop for SC lattices and ≈64 s per loop for FCC lattices. Unit-cell size (25–50 mm) had negligible influence on total winding time.

The effective winding angle relative to the horizontal plane was maintained between 35° and 55°, with an average of ≈45°, depending on lattice topology and local fiber trajectory. This orientation balanced vertical load transfer with lateral confinement while minimizing curvature at node transitions. Post-process image analysis confirmed angular deviations within ±2° across specimens.

Node engagement and overlap control

At lattice nodes, limited overlap between adjacent rovings was unavoidable. The measured overlap thickness was ≈0.48 mm, consistent with the flattened profile of a dry 24K bundle under light tension. In Type A supports, overlap occurred along curved wrap zones around 3 mm nodes; in Type B clip-lock systems, fibers were constrained within a nominal 0.4 mm gap. These controlled overlaps provided sufficient frictional engagement to prevent slippage during subsequent passes and impregnation, without inducing resin-rich regions or fiber damage.

Moderate helical twist (≈10°–20°) was observed along the roving due to directional changes in the three-dimensional path. This twist was stable within the selected tension range and did not lead to local slack or distortion after curing.

Together, these parameters define a mechanically stable winding window that preserves fiber straightness, suppresses relaxation, and ensures consistent geometry across specimens. While tension control in this study was achieved manually, the defined ranges are compatible with servo-controlled or load-cell–based feedback systems for future automated implementations.

S2: Fabrication Reproducibility

To assess fabrication reproducibility, ten nominally identical 37-SC-B-2 lattices were fabricated under identical winding, impregnation, and curing conditions. PLA support cores were demolded manually after 12 h of curing (green-cure stage), prior to full cure, to evaluate whether early support removal introduced additional variability.

The resulting specimens exhibited consistent geometry and mechanical response. Peak compressive force averaged 1384.5 ± 160.5 N (±11.6%), while the strength-to-weight ratio averaged 454 ± 38 N g^{-1} (±8.3%). No statistical outliers were observed, with all values falling within ±2σ of the mean. Individual specimen masses ranged from 2.71 to 3.56 g, reflecting minor variations in fiber content and resin uptake inherent to manual fabrication.

Despite this mass variation, force–displacement responses were highly consistent, with comparable initial stiffness, peak load, and post-buckling plateau behavior across all specimens (Supplementary Fig. 1). Peak force values ranged from 1184 to 1645 N, corresponding to a spread of ~28% in strength-to-weight ratio between the lowest and highest specimens, which is within the expected variability for manual winding processes.

Overall, these results demonstrate that continuity-governed lattice performance is robust to fabrication variability, and that the mechanical trends reported in the main text are reproducible under controlled manual fabrication conditions.

S3: Void Morphology and Content Analysis

Void morphology and distribution were examined to assess impregnation quality in the manually wound CFRP lattices. Imaging was performed using an upright metallurgical microscope (BX53MTRF, Olympus, Japan) equipped with motorized focus stacking to obtain high-depth-of-field images of composite cross-sections with uneven topography.

Individual CFRP struts were sectioned using high-strength composite shears to avoid fiber pull-out or interlaminar damage. As illustrated in Supplementary Fig. 2, cross-sections were prepared in two orientations: (i) perpendicular to the winding direction (horizontal sections) and (ii) parallel to the winding direction (vertical sections), the latter obtained by progressive surface grinding until the internal cross-plane was exposed. All surfaces were polished using SiC abrasive papers up to 2000 grit to achieve optical flatness and minimize polishing-induced artifacts.

Images were acquired under identical illumination and magnification conditions, converted to 8-bit grayscale, and binarized in ImageJ using the Triangle thresholding method. Void regions were identified as white pixels, and the areal void fraction was calculated as the ratio of void pixels to total pixels. Five independent fields of view were analyzed per specimen for each orientation, and mean values with standard deviations were computed.

The measured void fraction was 3.70 ± 0.55 vol% for horizontal sections and 1.79 ± 0.20 vol% for vertical sections, yielding an overall average void content of 2.75 ± 1.12 vol%. The higher void fraction observed in horizontal sections is attributed to localized air entrapment or resin starvation between adjacent tows, where curvature and overlap partially restrict resin flow. In contrast, vertical sections aligned with the fiber direction exhibited fewer discontinuities, indicating effective capillary-driven impregnation along the rovings.

When benchmarked against reported values for conventional CFRP fabrication routes, the measured void content indicates high consolidation efficiency. Manual hand lay-up typically exhibits void contents of ~10–25 vol%, vacuum-bagged laminates ~2–6 vol%, and filament-wound composites ~1–5 vol%. The present value therefore approaches the lower bound of filament-wound systems, despite the absence of autoclave processing or external compaction pressure.

S4: Shortest Path Winding Algorithm

The shortest-path strategy consisted of four integrated stages: lattice graph construction, path reconstruction, genetic-algorithm optimization, and modular segmentation $G = (V, E)$, where the vertices V corresponded to lattice nodes and the edges E represented physical struts. Unit cells of the SC, or FCC types were generated by translating a canonical cubic cell with eight labeled nodes (A–H). Each translation vector shifted these node positions, producing unique identifiers such as A_1_0_2, which recorded both the label and spatial coordinates. Edges were added according to lattice type: SC cells contained only cube edges, FCC cells included twelve face diagonals, and BCC cells included four body diagonals. Weights were assigned based on geometric length, with cube edges weighted as $w = 1$, face diagonals weighted as $w = \sqrt{2}$, and body diagonals weighted as $\sqrt{3}$. If multiple unit cells contributed the same physical strut, the edge was stored once, but tagged with all contributing unit-cell indices. This guaranteed that every physical strut was represented uniquely, and the completed graph therefore contained vertex attributes for spatial position and edge attributes for geometric weight and unit-cell membership.

Once the lattice graph was defined, a path reconstruction procedure was required to convert an arbitrary edge ordering into a valid continuous traversal. The routine began by seeding the path with the two endpoints of the first edge. For each subsequent edge, if the current endpoint already matched one of its nodes, the path was extended directly. If no match was present, the algorithm computed the shortest connector path from the current endpoint to one of the candidate nodes using Dijkstra's method [37], inserted this connector into the trail,

$$L_{path} = \sum_{(i,j)\in\wp} w_{ij} \tag{S2}$$

where $\wp$ is the ordered sequence of struts and connectors in the reconstructed path, and $w_{ij}$is the geometric weight of each edge (*i, j*). A cleanup stage was then applied to remove duplicate consecutive nodes created by connector insertion, producing one feasible continuous winding path consistent with the true geometry of the lattice.

To identify the most efficient ordering of edges, a genetic algorithm was implemented. Each individual in the population was encoded as a permutation of the edges, and its fitness was defined as the reconstructed path length,

$$f(individual) = L_{path} \tag{S3}$$

so that lower values indicated more efficient windings. The GA was initialized with 50 random permutations and evolved over 200 generations using the DEAP framework. Ordered crossover preserved promising subsequences of edges, shuffle mutation introduced diversity, and tournament selection balanced exploration with selection pressure. Crossover and mutation probabilities were set to 0.7 and 0.3, respectively. Over successive generations, the GA consistently converged toward edge orderings that minimized the total path length.

Following optimization, the continuous path was segmented into fabrication-ready modules. The segmentation was based on unit-cell membership tags assigned to edges during graph construction. Consecutive edges that shared the same unit-cell identifier were grouped into a single module, while transitions across overlapping cells created separate groups. This modular breakdown aligned the winding sequence with the physical assembly of unit cells, thereby facilitating both fabrication and structural analysis.

Visualization showed struts in black, nodes in red, and final continuous paths in green with traversal order annotated. Console outputs included optimized length, edge sequence, and segmentation list.

S5: Multi-Cube Assembly

Following single-cell evaluation, multi-cellular CFRP lattices were fabricated to assess modular scalability. Type A BCC cores and nodes were manually stacked to form assemblies up to 2×2×2 (eight cells) (Supplementary Fig. 4c). At each step, unit cells were aligned in three spatial directions, and shared nodes were wound with additional fiber passes to maintain continuity across the assembly.

To guide fabrication, shortest-path algorithms (Supplementary Fig. 4a,b; Supplementary Method S1) were used to generate continuous winding routes. The path density increased rapidly with cube size—for example, a single FCC cell required ~100 weighted steps, while the FCC 2×2×2 expanded beyond 260. Without algorithmic planning, redundant overlaps and slack loops accumulated quickly, highlighting the importance of automated optimization for practical scaling.

Fabrication results reflected these trends. The SC-25-2 cube (Supplementary Fig. 4d) was wound cleanly with uniform geometry, confirming that low-node-density SC assemblies remain tractable even by manual methods. The FCC-25-1 cube (Supplementary Fig. 4e), however, exhibited irregular winding, resin-rich defects, and visible distortion. These issues were attributed primarily to the difficulty of manually routing fibers through the highly congested interior of small FCC cells, rather than a fundamental limitation of the architecture. Larger FCC assemblies performed more favorably: an FCC-50-1 hollow cube (Supplementary Fig. 4f) produced uniform winding after support removal, as omitting interior diagonals simplified the path sequence. Although mechanically weaker, such hollow FCC frameworks demonstrate a viable strategy for manufacturable, large-scale assemblies where geometric openness and regularity are prioritized.

S6: Extended Derivation of Continuity Factor

Directional continuity is expressed as:

$$\eta_{\mathrm{eff}}(n) = \sum_{i\in\{x,y,z\}} (n \cdot e_i)^2\, \eta_i \tag{S4}$$

where η_i represents the continuity efficiency along axis i. To avoid the unphysical case where discontinuous fibers, predict zero strength, a matrix backstop is introduced:

$$\eta_i = \eta_{f,i} + \left(\frac{E_m}{E_c^{(i)}}\right)(1 - \eta_{f,i}) \tag{S5}$$

where $\eta_{f,i}$ is the fiber-network continuity. This guarantees that even in the absence of continuous fibers, the matrix contribution provides a finite baseline strength. The quantity $\eta_{f,i}$ depends on architecture and fabrication. Simple estimators can be derived for each class. For continuous fiber winding, only start/end terminations reduce continuity. If N_i tows span direction i with average node quality $\bar{\kappa}_i$,

$$\eta_{f,i} \approx \bar{\kappa}_i\left(1 - \frac{2}{N_i}\right) \tag{S6}$$

Node congestion modifies this through a fixity factor γ that accounts for the loss of rotational stiffness at overlaps. For SC lattices (3 overlaps per node), $\gamma \approx 0.9{-}1.0$. For FCC lattices (6 overlaps), γ is slightly lower ($\sim$0.8−0.9) but still favorable due to diagonal stabilization. For BCC lattices (8 overlaps at the center node), congestion strongly reduces fixity ($\gamma < 0.7$), making them less efficient despite higher coordination number. Applying γ yields the effective continuity

$$\eta_{f,i} \leftarrow \gamma \eta_{f,i} \tag{S7}$$

For laminates, continuity is high in-plane but reduced in the Z-direction. With edge cut width δ_{cut} , panel width W, ply thickness t_p, shear-lag length l_s, and number of Z-reinforcements N_Z:

$$\eta_{f,x} \approx \eta_{f,y} \approx 1 - \frac{\delta_{\text{cut}}}{W}, \qquad \eta_{f,z} \approx \kappa_z \frac{t_p}{t_p + l_s} \frac{N_z}{N_x + N_y + N_z} \tag{S8}$$

Thus, XY continuity remains near unity, while Z continuity is typically 0.1–0.5 without stitching or pins.

For short-fiber composites, Shear-lag theory [38] provides a natural form. With average fiber length $\bar{l}$, critical length $l_c = \sigma_f d/(2\tau)$, and orientation factor $f_2^{(i)} = \langle cos^2 \theta_i \rangle$:

$$\eta_{f,i} \approx \left(1 - e^{-\bar{l}/l_c}\right) f_2^{(i)} \tag{S9}$$

Values increase with fiber aspect ratio and alignment but remain far below unity. For particulate composites, no continuous fiber paths exist, so $\eta_{f,i} \approx 0$. Continuity reduces to the matrix backstop:

$$\eta_i \approx \frac{E_m}{E_c^{(i)}} \tag{S10}$$

Eq. S3-S9 establish continuity as a measurable, architecture-dependent parameter. Continuous winding of SC and FCC lattices maximizes η, with SC favored for minimal node congestion and FCC providing isotropy with only a modest reduction in continuity. BCC and other congested topologies suffer from reduced fixity and lower η. Laminates retain high in-plane continuity but low through-thickness connectivity, while discontinuous systems occupy much lower bands. Incorporating these effects, the Gibson–Ashby framework transforms from a pair of scaling curves into performance bands bounded by continuity. As illustrated in Supplementary Fig. 5c, lattices with poor continuity are confined to the lower bands, while continuous winding ($\eta \rightarrow 1$) allows SC and FCC lattices to approach the theoretical limits. Supplementary Table 3 summarizes the continuity estimates by material class.

S7: Theoretical Buckling of Single Columns

The cross-sectional properties of each wound CFRP tow were first determined experimentally. Individual columns were cut at mid-length, photographed alongside a calibrated ruler, and analyzed in ImageJ using polygonal tracing and pixel-to-length conversion to obtain the true cross-sectional area A. This direct measurement was used in all subsequent calculations, ensuring that geometric variability between tows was accurately captured.

The compressive load capacity of single columns was then estimated using classical Euler buckling theory, with successive refinements to account for material nonlinearity, shear flexibility, and

fabrication imperfections. These calculations provided the baseline predictions against which the more complex lattice models were later compared.

The axial modulus of a unidirectional tow was calculated using the rule of mixtures based on the measured fiber volume fraction V_f. The longitudinal modulus is therefore

$$E_c = E_f V_f + E_m\left(1 - V_f\right) \tag{S10}$$

where the fiber modulus E_f was taken as 250 GPa, and the matrix modulus E_m was taken as 3.17 GPa for epoxy and 3.2 GPa for vitrimer. When available, the measured initial tangent modulus in compression from the 0–0.3 % strain region was used in place of the rule-of-mixtures value, as this better captures the stiffness of short columns that fail by material crushing rather than by global buckling.

The shear modulus of the composite tow was also required in order to capture shear flexibility in short and intermediate columns. This property was estimated using the Halpin–Tsai model:

$$\eta = \frac{\frac{G_f}{G_m} - 1}{\frac{G_f}{G_m} + \xi}, \qquad G_{12} = G_m \frac{1 + \xi \eta V_f}{1 - \eta V_f} \tag{S11}$$

where the fiber shear modulus transverse to the loading direction was taken as G_f = 15 GPa, the matrix shear modulus was computed as $G_m = E_m/[2(1 + \upsilon_m)]$, with υ_m = 0.35, and the fitting parameter was set as $\xi = 1$. The geometric properties of each tow cross-section were determined from the measured cross-sectional area A. An equivalent diameter was defined as

$$d = \sqrt{\frac{4A}{\pi}}, \qquad I = \frac{\pi d^4}{64}, \qquad r_g = \sqrt{\frac{I}{A}} \tag{S12}$$

where I is the second moment of area and r_g is the radius of gyration. The slenderness ratio was then calculated as

$$\lambda = \frac{KL}{r_g} \tag{S13}$$

where L is the gauge length and K is the end-fixity parameter. A value of K between 0.7 and 1.0 was appropriate, and for each tow family a representative K was selected by fitting the slenderest specimen (largest L) to ensure that the Euler prediction matched the experimental buckling stress.

For slender columns, the Euler stress is

$$\sigma_E = \frac{\pi^2 E_c}{\lambda^2} = \frac{\pi^2 E_c I}{A(KL)^2} \tag{S14}$$

However, this ideal model does not account for shear deformation, which becomes relevant at intermediate slenderness. To correct this, the Timoshenko–Engesser shear-flexible column model was applied. The corresponding load is

$$P_T = \frac{P_E}{1 + \frac{\pi^2 E_c I}{\kappa G_{12} A (KL)^2}}, \qquad P_E = \frac{\pi^2 E_c I}{(KL)^2} \tag{S15}$$

where $\kappa = 0.9$ for circular cross-sections. This correction reduces the predicted buckling load when shear flexibility lowers column stiffness. At short slenderness ratios, columns do not buckle but instead fail by crushing or kink-band formation. To capture this inelastic regime, the Johnson–Ostenfeld transition was used. The material limit strength σ_m was taken as the experimentally measured short-column strength at $L = 30$. A transition slenderness λ_t was defined as

$$\lambda_t = 2\pi \sqrt{\frac{E_c}{\sigma_m}} \tag{S16}$$

For columns with $\lambda \leq \lambda_t$, the Johnson stress was calculated as

$$\sigma_J = \sigma_m \left(1 - \frac{\sigma_m}{4\pi^2 E_c} \lambda^2\right) \tag{S17}$$

While for $\lambda > \lambda_t$, the Euler stress σ_E was retained. Finally, imperfections due to initial crookedness, end-compliance in the fixtures, and small misalignments were incorporated through a single empirical knock-down factor χ. This factor was determined for each tow family by fitting an intermediate-length specimen (e.g. $L = 65$ mm):

$$\chi = \left. \frac{\sigma_{exp}}{min(\sigma_J, \sigma_T)} \right|_{L=65mm}, \qquad 0 < \chi \leq 1 \tag{S18}$$

The final predicted column stress and load were therefore

$$\sigma_{pred} = \chi \, min(\sigma_J, \sigma_T), \qquad P_{theory} = A\sigma_{pred} \tag{S19}$$

This framework allows a smooth transition across three regimes: crushing of short columns, Johnson-type inelastic buckling at intermediate slenderness, and Euler/Timoshenko global buckling at high slenderness. It provides a consistent baseline for comparing theoretical and experimental responses of single CFRP columns.

S8: Theoretical Buckling of Lattices

The theoretical compressive strength of the lattice structures was also estimated by extending the single-column framework to account for the multiple interacting members within each unit cell.

While the basic instability mechanism remained dominated by the buckling of vertical struts, the presence of horizontal and diagonal members introduced additional load-sharing and restraint effects that were incorporated into the analysis.

The total effective cross-sectional area of the lattice was first determined experimentally from the measured mass of the specimen and the known densities of PLA and CFRP. This effective area was then distributed among the individual members based on lattice geometry and loop number. For SC lattices, where only vertical and horizontal members exist, the average member area was calculated as

$$A_m = \frac{A_{tot}}{n_v + n_h} \tag{S20}$$

where n_v and n_h are the numbers of vertical and horizontal members, respectively. For FCC lattices, diagonal members were also included, and because each diagonal contributes both a horizontal and vertical component, their load-carrying area was doubled:

$$A_m = \frac{A_{tot}}{n_v + n_h + \sqrt{2}n_d} \tag{S21}$$

where n_d is the number of diagonal struts. Once the average member area was established, each member was idealized as a cylinder of equivalent area. The effective diameter and second moment of area were calculated from

$$d_m = \sqrt{\frac{4A_m}{\pi}}, \qquad I_m = \frac{\pi d_m^4}{64} \tag{S22}$$

Although the end conditions of lattice struts differ from isolated single columns, an effective boundary condition factor was defined to approximate their restraint. The nominal end-fixity parameter was taken as K = 1.2, intermediate between pinned and fixed ends. However, in cubic frameworks the presence of horizontal connections provides additional elastic restraint. A first-order Rayleigh–Ritz approximation was used to estimate this stiffening effect, yielding a reduction factor β:

$$\beta = \sqrt{\frac{12E_cI_m}{G_{12}A_mL^2} + 1} \tag{S23}$$

where L is the vertical member length, E_c and G_{12} are the effective moduli previously defined in Eq. 9 and Eq. 10. For CFRP with $G/E \approx 0.05$, the reduction factor was approximately 1.15. The effective boundary condition factor was then defined as

$$K_{eff} = \frac{K}{\beta} \approx \frac{1.2}{1.15} = 1.04 \tag{S24}$$

Using this effective boundary condition, the Euler buckling load for vertical members was calculated as

$$P_e = \frac{\pi^2 E_c I_m}{\left(K_{eff} L\right)^2} \tag{S25}$$

For diagonal members, only the vertical component of their load-bearing capacity was considered. This was expressed as

$$P_{e,d}^V = \frac{\pi^2 E_c I_m}{\left(K_{eff} L_d\right)^2} cos45° \tag{S26}$$

where L_d represent the diagonal length. Manufacturing imperfections, such as fiber waviness and defects inherent in the fabrication process, significantly affect load-bearing capacity. Typically, an imperfection factor (ϕ) ranging from 0.3 to 0.5 is employed in the literature to account for these manufacturing deviations. Thus, the imperfection factors applied in this study are:

$$\phi = \begin{cases} 0.3 + 0.05l \ \ (SCC) \\ 0.3 + 0.075l \ (FCC) \end{cases} \tag{S27}$$

Finally, the total theoretical lattice capacity was determined by summing the contributions of the vertical and diagonal members and applying the imperfection factor:

$$P_{th} = \phi\left(n_v P_e + n_d P_{e,d}^V\right) \tag{S28}$$

This formulation therefore integrates the single-column buckling model, corrected for shear flexibility and imperfections, with the member count and geometric arrangement unique to each lattice type. The result provides a theoretical estimate of the critical compressive load that can be directly compared against experimental measurements for both SC and FCC lattices with different loop numbers.

S9: Cyclic Compression Testing

Cyclic compression tests were conducted to evaluate the mechanical stability and hysteresis behavior of continuous CFRP lattices under repeated loading. Two representative architectures were selected: a SC lattice with 50-mm unit cells fabricated using Type-B nodes and a single winding loop, which fails predominantly by elastic buckling, and a simple cubic lattice with 37-mm unit cells fabricated using Type-B nodes and two winding loops, which exhibits kink-crushing–dominated collapse under monotonic compression.

Displacement-controlled cyclic tests were performed for 24 consecutive loading–unloading cycles. The maximum displacement was fixed at 50% of the monotonic deformation at peak load, corresponding to approximately 0.9–1.0 mm for the 50-mm, single-loop SC lattice and 1.8 mm for the 37-mm, two-loop SC lattice. This displacement amplitude was selected to impose a severe yet

non-catastrophic deformation, consistent with cyclic compression protocols commonly used for cellular materials.

For the 50-mm, single-loop SC lattice, cyclic loading produced a gradual increase in apparent stiffness across cycles, with no measurable permanent height reduction. This pseudo-hardening behavior is attributed to the stabilization of initially buckled members into repeatable load paths without progressive damage accumulation. In contrast, the 37-mm, two-loop SC lattice exhibited a rapid stiffness increase during early cycles, followed by gradual softening and controlled height reduction, consistent with progressive kink-crushing and densification. Despite this densification, the lattice retained substantially higher stiffness and load-bearing capacity throughout cycling.

To further assess durability under service-relevant conditions, complementary force-controlled cyclic tests were performed for 50 cycles using a peak load of 40 kgf (≈390 N) with a 5 kgf preload to eliminate initial compliance. Under force-controlled cycling, both lattice architectures exhibited stable hysteresis loops and full dimensional recovery, with no evidence of catastrophic collapse or runaway damage.

Together, the displacement- and force-controlled cyclic tests demonstrate that continuity-preserving CFRP lattices tolerate repeated mechanical loading through stable buckling and kink-crushing mechanisms rather than brittle fracture. These results confirm the suitability of such architectures for applications requiring repetitive load bearing, including lightweight structural frames, robotic components, and energy-absorbing systems.

S10: Scalability of Hover Endurance

To assess whether the endurance gains of lattice frames persist with vehicle size, hover time was modeled as a function of motor-to-motor span L. Two cases were considered: (i) fixed propellers (5-inch × 4 rotors, MT2204-class motors, 3S-2200 mAh pack) and (ii) constant disk loading with fixed battery capacity.

For fixed rotors, the total disk area A_{tot} remains constant, so induced power dominates:

$$P_{ind} = \frac{W^{\frac{3}{2}}}{2\rho A_{tot}}, \qquad t \approx \frac{C}{W^{\frac{3}{2}}}, \qquad C = \frac{\eta E_{batt}}{2\rho A_{tot}} \tag{S29}$$

where $W = mg$ is the system weight, E_{batt} the usable battery energy, and η captures non-induced losses. The constant C was calibrated for each airframe using the 250 mm experimental baseline. System mass was decomposed into electronics and frame contributions:

$$W(L) = m_{elec} + m_{frame}(L) \tag{S30}$$

with m_{elec} taken as span-invariant. To enforce stiffness requirements, the frame was expressed as a span-independent hub plus arms that must thicken with span to bound deflections. With measured scaling $EI \propto h^{4.7}$, constant tip deflection requires

$$m_{arm}(L) \propto \left(\frac{L}{L_0}\right)^{1.638} \tag{S31}$$

So that

$$m_{frame}(L) = m_{hub} + m_{arm,0}\left(\frac{L}{L_0}\right)^{1.638} \tag{S32}$$

where m_{hub} and $m_{arm,0}$ are calibrated at $L_0 = 250$ mm. Substituting Eq. S27-29 into Eq. S26 yields the endurance prediction curves.

In the fixed-prop case (Fig. 4j), endurance falls monotonically with increasing span as system mass rises while available thrust area remains constant. However, the relative margin of the lattice frames persists: CFRL2 consistently maintains the highest endurance, while solid CFRP lies between the nylon and lattice variants.

In the constant-disk-loading case (Fig. 4k), propeller diameter is resized to hold $DL = DL_{250}$ while the same battery is retained. Endurance still decreases with span due to rising system weight, but for the CFRL2 baseline this requires propeller diameter to grow from ~5.0 in at 250 mm to ~5.7 in at 1000 mm (curve shown on right axis). Again, CFRL2 sustains the highest endurance across the size range, confirming that lattice efficiency benefits extend beyond micro-drones to larger platforms constrained by structural stiffness.

S11: Peak Load Measurements for Robotic Arm

A robotic arm originally fabricated from PLA was redesigned using CFRP lattice arms to demonstrate the performance advantages of modular 3D wound lattices. The baseline PLA arms weighed 145 g in total (Supplementary Fig. 9a), while the CFRP replacements weighed only 67.1 g (Supplementary Fig. 9c), corresponding to a 53.7% reduction in mass. The CFRP design used Type-B ribbed cores specifically adapted for the forearm link (Supplementary Fig. 9b), enabling continuous winding along the full span. Each arm measured 250 mm in length, giving a 500 mm total reach, and both versions were actuated by Dynamixel XM540-W270-T servos.

Peak end load increased from 15.94 N for the PLA arms (Supplementary Fig. 9d) to 17.49 N for the CFRP lattice arms (Supplementary Fig. 9e), yielding a 9.7% higher load capacity despite halving the mass. This improvement arises partly from reduced gravitational torque at the joints. For a uniform link of mass m and length L, the gravitational torque is

$$\tau_g = \frac{mgL}{2} \tag{S33}$$

and for the two-link system including the interlink motor,

$$\tau_{g,base} = \frac{m_1 gL}{2} + m^2 g\left(L + \frac{L}{2}\right) + m_{motor} gL \tag{S34}$$

Substituting measured values showed that base-joint torque dropped by 22.7%, while mid-joint torque fell by 44.8%. These reductions directly translate into lower actuator current through

$$\eta = \frac{F_{peak}}{m} \tag{S35}$$

which increased from 110 $N \cdot kg^{-1}$ for PLA to 261 $N \cdot kg^{-1}$ for CFRP, a 137 % improvement. In summary, the CFRP lattice arms not only reduced mass and torque demands but also delivered higher load capacity and more than doubled structural efficiency, clearly outperforming the PLA baseline.

S12: Miniature Aircraft Frame Assembly

To explore the adaptability of continuous CFRP lattice winding beyond cubic cells, a miniature aircraft prototype was fabricated (Supplementary Fig. 11a). Unlike drones or robotic arms, the aircraft frame imposed additional geometric and aerodynamic constraints, requiring thin lifting surfaces, lightweight stabilizers, and a streamlined fuselage. This motivated the incorporation of alternative lattice types—square grids and triangular prisms—into the winding pipeline.

A planar four-node square lattice (A–B–C–D) was adopted as a base cell to construct lightweight 2D assemblies for the tail section. Because all nodes lay in the same plane, shortest paths naturally reduced to Hamiltonian cycles with minimal redundancy, enabling efficient winding without connectors. Supplementary Fig. 10a illustrates optimized paths for 1×2 and 2×2 assemblies. The 2D square base cell was defined as follows:

```
base_nodes = ["A","B","C","D"]
base_positions = { "A":(0,0,0), "B":(1,0,0), "C":(1,1,0), "D":(0,1,0) }
base_edges = [("A","B"),("B","C"),("C","D"),("D","A")]
```

A six-node, nine-edge triangular prism lattice was introduced to approximate thin wing profiles. At the 1×1 scale, the optimal continuous winding path required 11 steps; for a 1×4 prism beam, the optimized path extended to 34 steps (Supplementary Fig. 10b). These prism modules provided stiffness while maintaining the slender aspect ratio required for airfoil geometries. The triangular-prism base cell was parameterized using six nodes and nine edges:

```
base_nodes = ["T1","T2","T3","B1","B2","B3"]
s3 = math.sqrt(3)/2
base_positions = {
    "T1":(0,0,0),"T2":(1,0,0),"T3":(0.5,s3,0),
    "B1":(0,0,1),"B2":(1,0,1),"B3":(0.5,s3,1)
}
base_edges = [
    ("T1","T2"),("T2","T3"),("T3","T1"),
    ("B1","B2"),("B2","B3"),("B3","B1"),
    ("T1","B1"),("T2","B2"),("T3","B3")
]
```

A Clark-Y airfoil cross-section was used as the wing profile (Supplementary Fig. 11e). PLA scaffolds were first printed in triangular-prism form to capture the 5 mm airfoil thickness, followed by continuous CFRP winding and resin impregnation. The resulting lattice preserved the aerodynamic profile with high geometric fidelity (Supplementary Fig. 11f,g).

The complete prototype integrated three topologies: (i) five SC cells for the fuselage (Supplementary Fig. 11b), (ii) four triangular-prism modules for the main wing (Supplementary Fig. 8d), and (iii) two square-grid units for the tail (Supplementary Fig. 11c). Their optimized path lengths were 87, 34, and 7 segments, respectively, yielding a total of 128 segments. The fully assembled 500 mm wingspan aircraft is shown in Supplementary Fig. 11h, weighing <50 g (≈0.06 g cm^{-3} relative density).

While SC lattices provided axial stiffness for the fuselage and prism modules reproduced thin wing sections, fabrication of small-scale wings remained challenging. Node clustering and near-coplanar struts caused local waviness and resin pooling, issues that would diminish at larger scales or with robotic winding.

This case study demonstrates that multiple lattice topologies can be fused into a single continuous CFRP frame. Although aerodynamic testing was not performed, the prototype illustrates the scalability of algorithmically optimized winding strategies toward aerodynamically relevant, lightweight aerospace structures.

Supplementary Figures

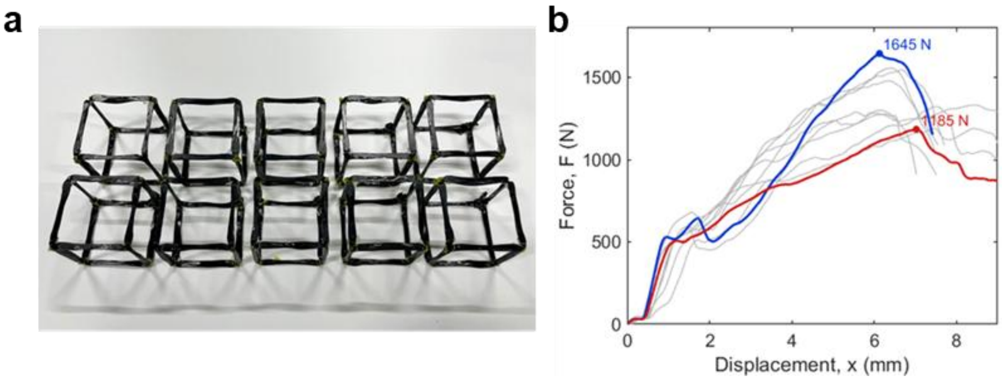

Supplementary Fig. 1. Fabrication reproducibility of 37-SC-B-2 lattices.
a, Ten nominally identical 37-SC-B-2 CFRP lattice specimens fabricated under identical winding and impregnation conditions. **b,** Corresponding force–displacement curves from uniaxial compression tests, showing consistent initial stiffness, peak load, and post-buckling plateau behavior across all specimens.

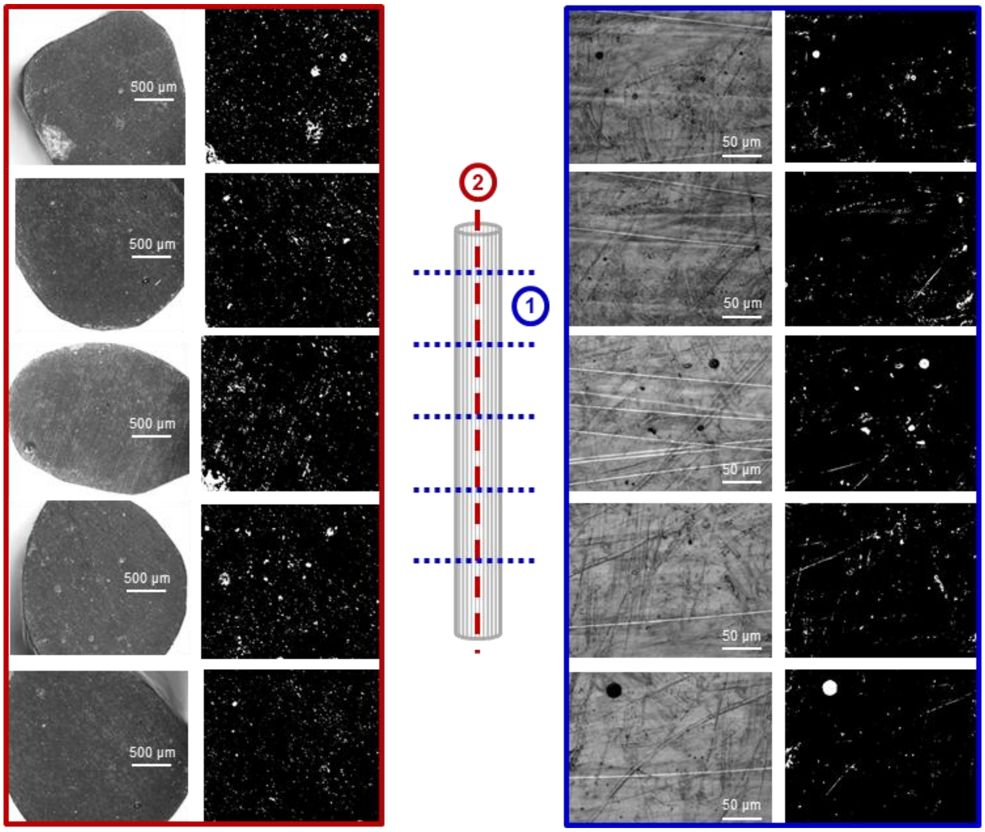

Supplementary Fig. 2. Preparation and image processing for void content analysis

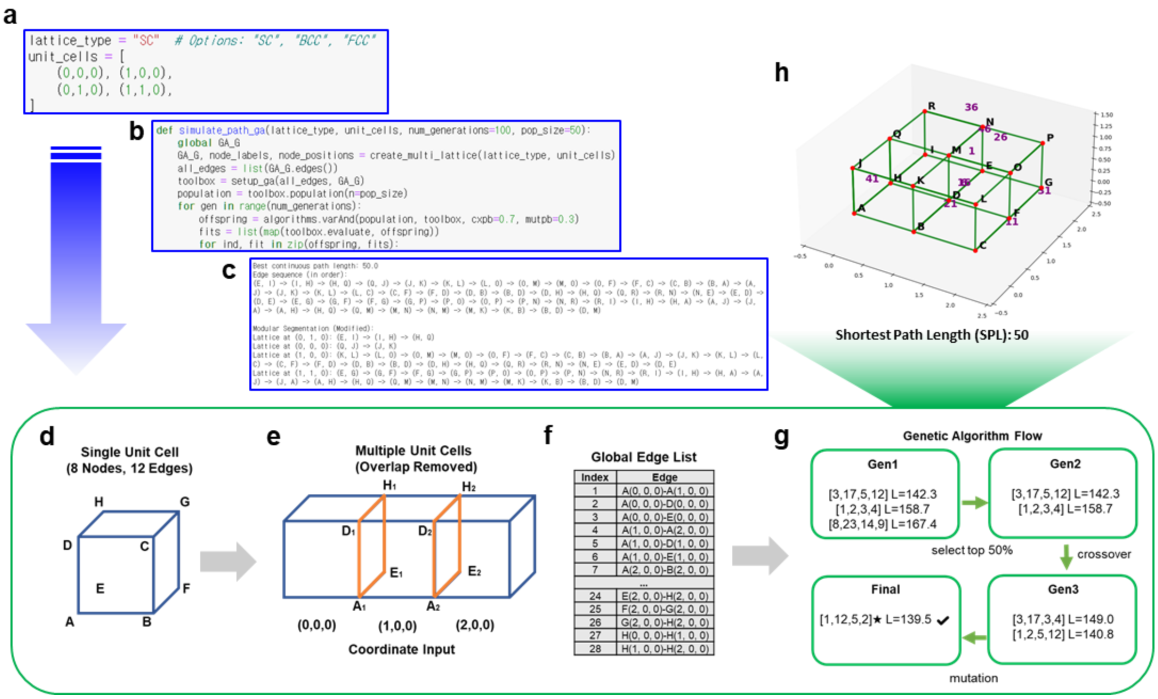

Supplementary Fig. 3. Workflow of shortest-path algorithm for continuous winding

a, Input lattice coordinates in 3D space. **b,** Genetic algorithm code snippet for optimization. **c,** Example output path with minimized length. **d,** Single SC cell generated. **e,** Three aligned SC cells with duplicate edges removed. **f,** Global edge list construction. **g,** Genetic algorithm workflow: selection, crossover, mutation. h, Final output graph with nodes renumbered for winding.

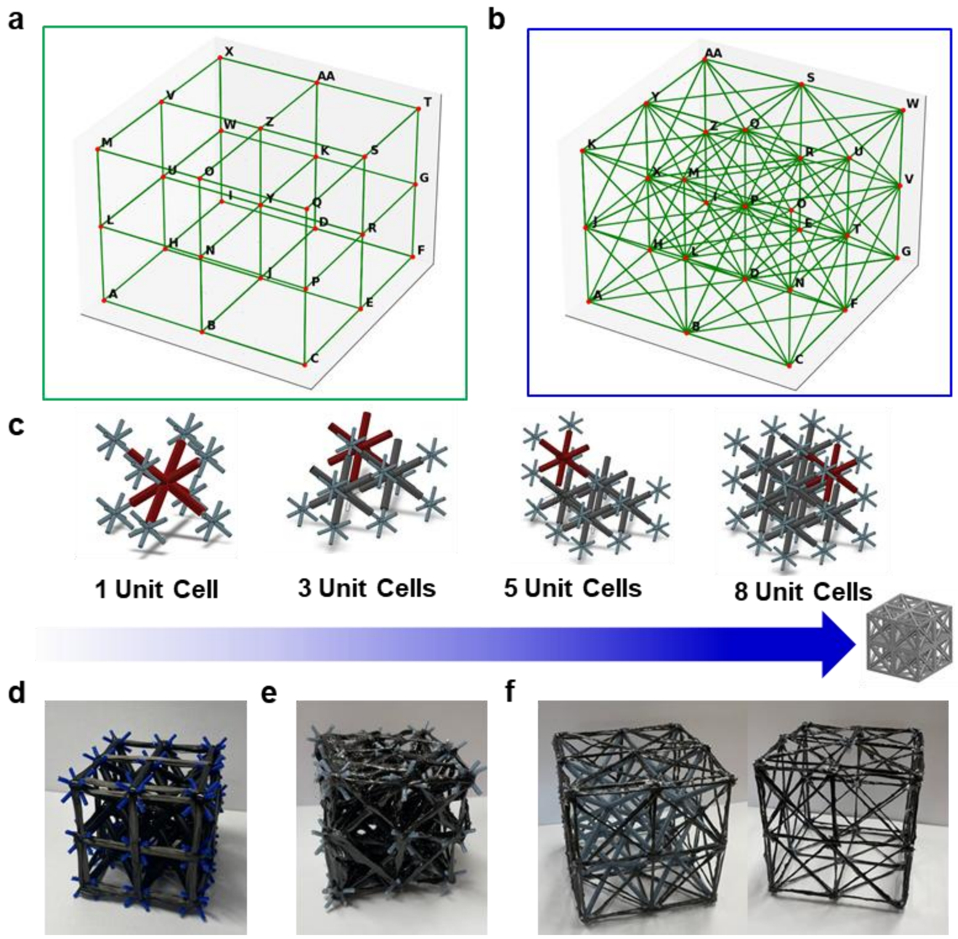

Supplementary Fig. 4. Multi-cube lattices and manufacturability.
a, Shortest-path output graph for SC 2×2×2 assembly. **b,** Shortest-path output graph for FCC 2×2×2 assembly. **c,** Core and node stacking sequence for multi-cube fabrication. **d,** Fabricated SC-25-2 lattice showing clean winding, compared with **e,** FCC-25-1 where excessive internal overlaps caused deformation and resin-rich defects. **f,** FCC-50-1 fabricated with hollow interior, shown before and after support removal; eliminating congested internal paths simplified winding and improved quality. These results demonstrate how tuning the shortest-path code to exclude inaccessible internals enables larger, more practical FCC assemblies.

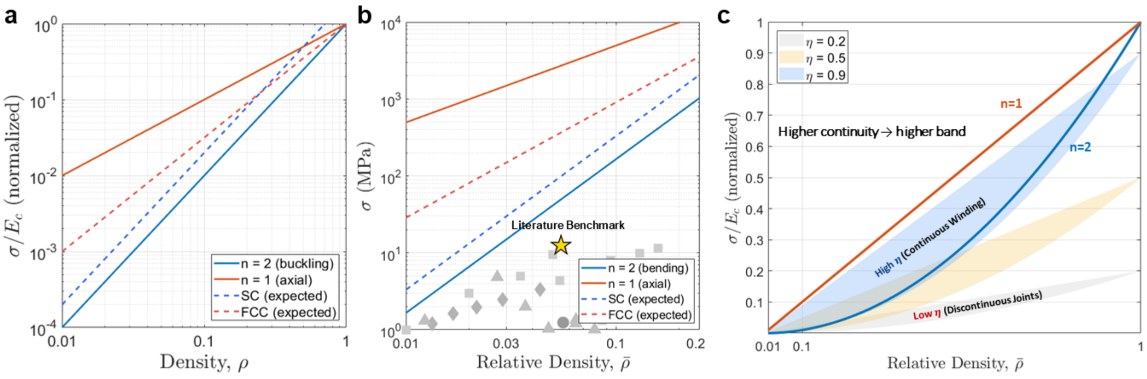

Supplementary Fig. 5. Gibson–Ashby scaling and continuity factor.
a, Expected placement of SC and FCC lattices within the classical Gibson–Ashby framework (n = 1, stretch; n = 2, buckling). **b,** Compressive strength vs relative density, showing prior mesoscale lattices and the new record regime. **c,** Extension of the framework with continuity factor η, where higher connectivity raises performance bands toward the theoretical limit.

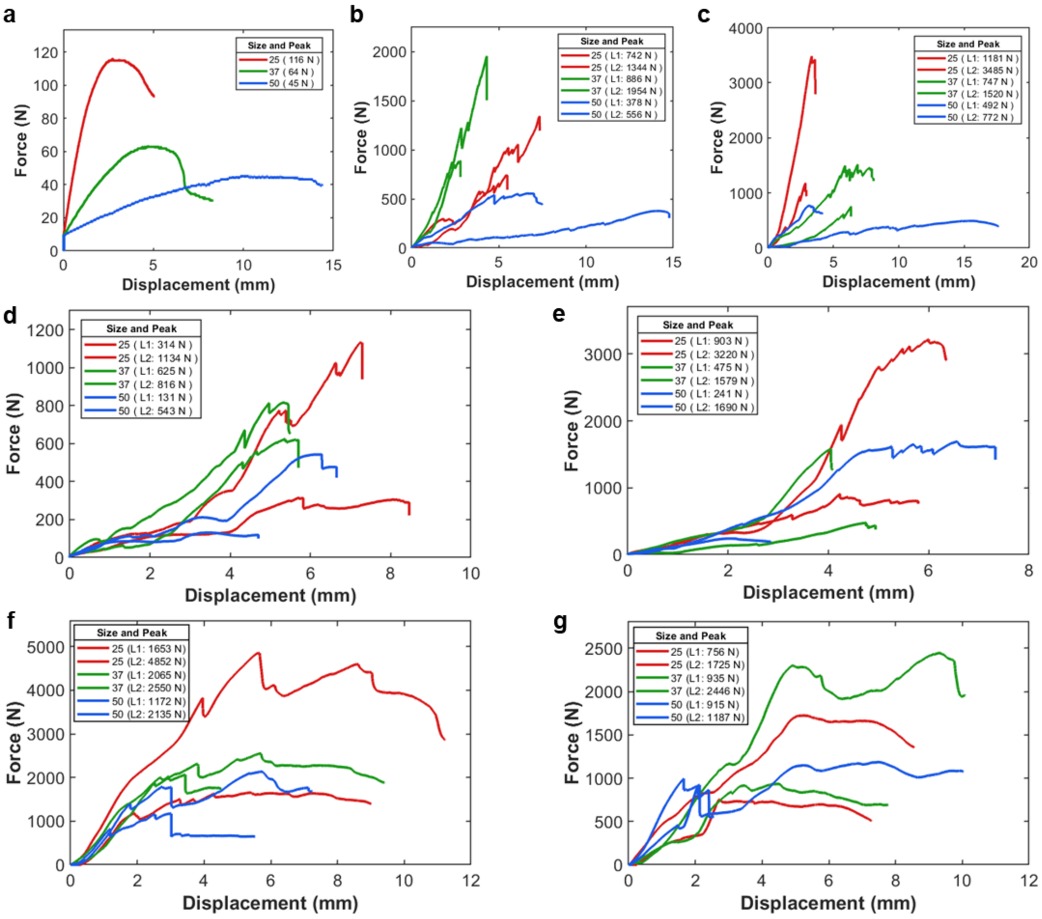

Supplementary Fig. 6. Force–displacement curves of CFRP lattices.
a, Type-A PLA BCC cores. **b,** SC lattices with BCC cores. **c,** FCC lattices with BCC cores. **d,** SC lattices (Type A). **e,** FCC lattices (Type A). **f,** SC lattices (Type B). **g,** FCC lattices (Type B).

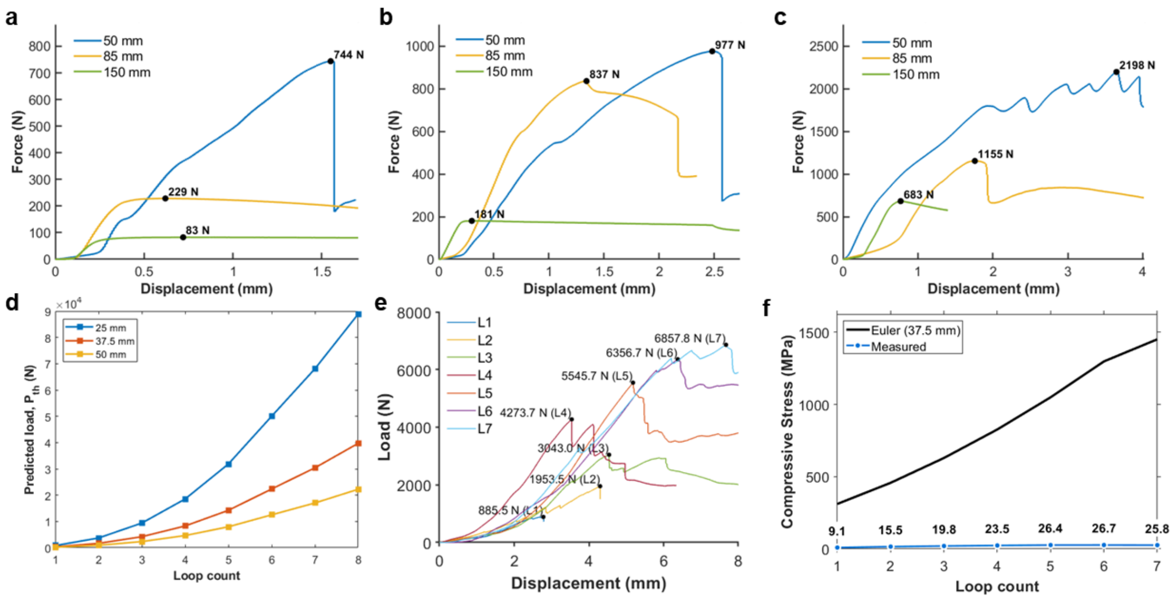

Supplementary Fig. 7. Compressive response of single columns and SC lattices.
a, Force–displacement curves for 50, 85, 150 mm columns (1 loop). **b,** Columns with 2 loops. **c,** Columns with 3 loops. **d,** Predicted Euler buckling loads for SC lattices**. e,** Experimental response of 37 mm SC lattices with 1–7 loops (single representative test per loop count). **f,** Euler-predicted vs. measured compressive stresses.

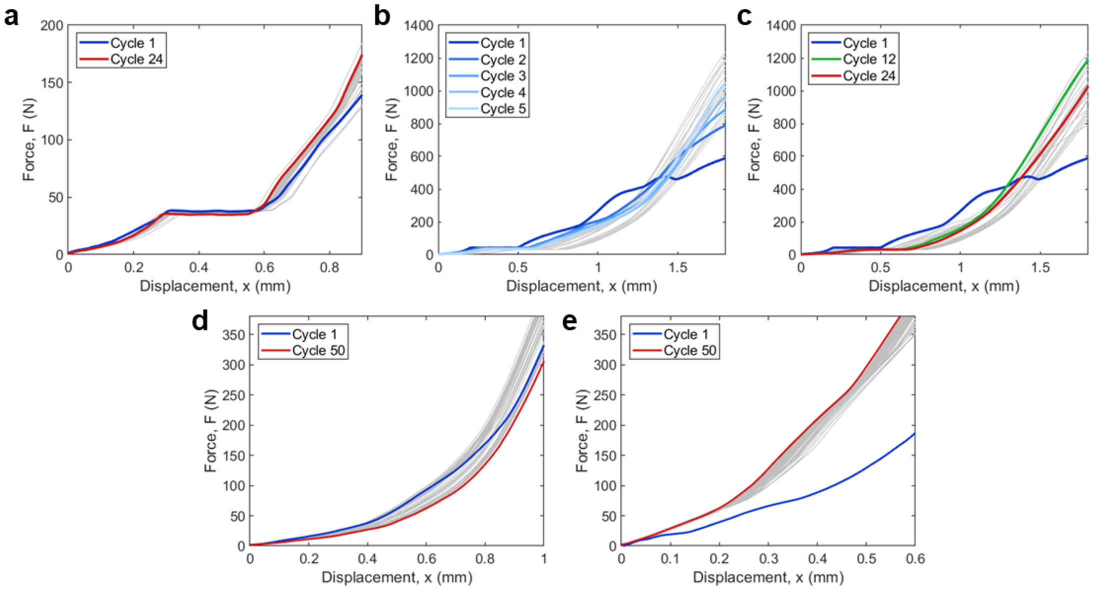

Supplementary Fig. 8. Cyclic compression behavior of continuous CFRP lattices
a, Displacement-controlled cycling of a buckling-dominated SC lattice, showing progressive stiffening without permanent height loss. **b,** Displacement-controlled cycling of a kink-crushing–dominated SC lattice, showing initial stiffening followed by controlled softening and densification. **c,** Representative hysteresis loops for the buckling-dominated lattice. **d,** Force-controlled cycling of a buckling-dominated SC lattice. **e,** Force-controlled cycling of kink-crushing-dominated SC lattice, showing stable hysteresis and full dimensional recovery.

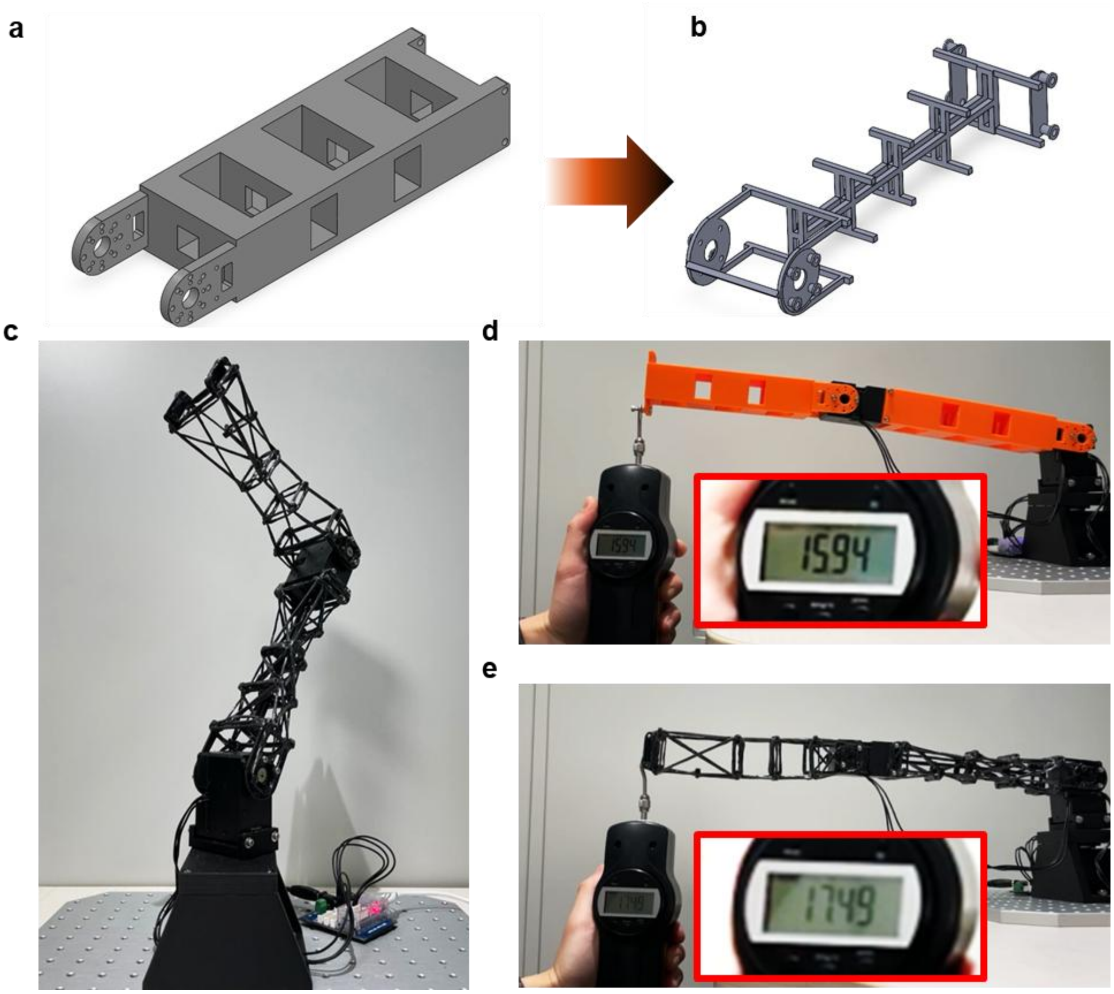

Supplementary Fig. 9. Robotic arm demonstrator.
a, Baseline PLA forearm link. **b,** Type-B ribbed core designed specifically for the robotic arm link, enabling continuous winding along the full span. **c,** Fabricated hybrid SC–FCC lattice link. **d–e,** Load tests: the PLA link lifted 15.94 N while the CFRP lattice link lifted 17.49 N, despite a 53.7% reduction in mass, demonstrating superior specific efficiency.

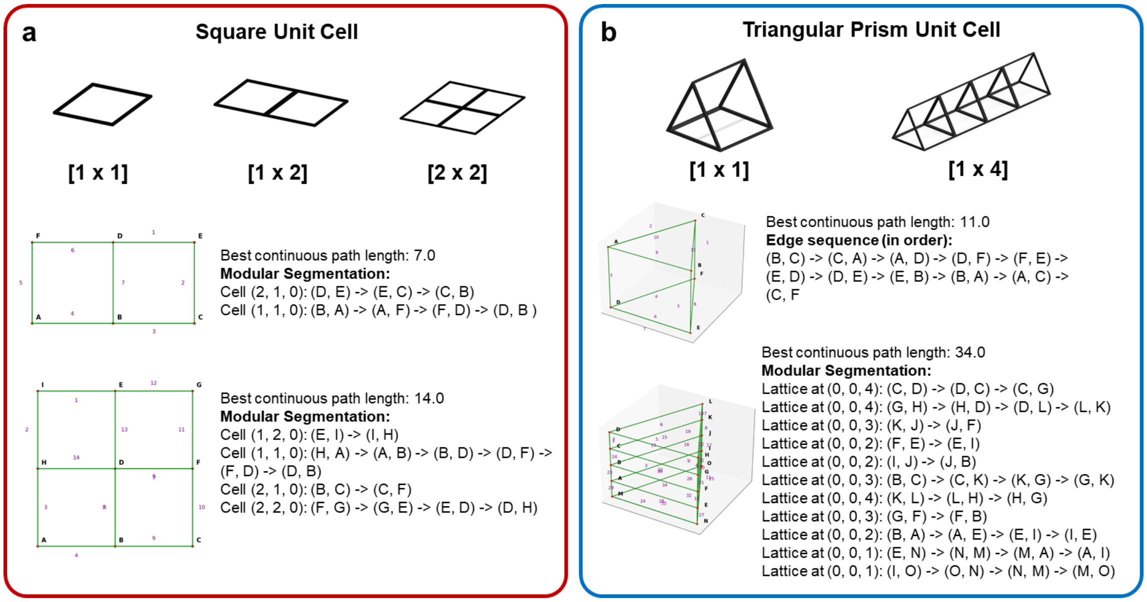

Supplementary Fig. 10. Shortest-path results for alternative unit cells.
a, 2D square lattice (1×2 and 2×2) with segmentation and paths. **b,** Triangular prism unit cell and 1×4 assembly, with edge sequences.

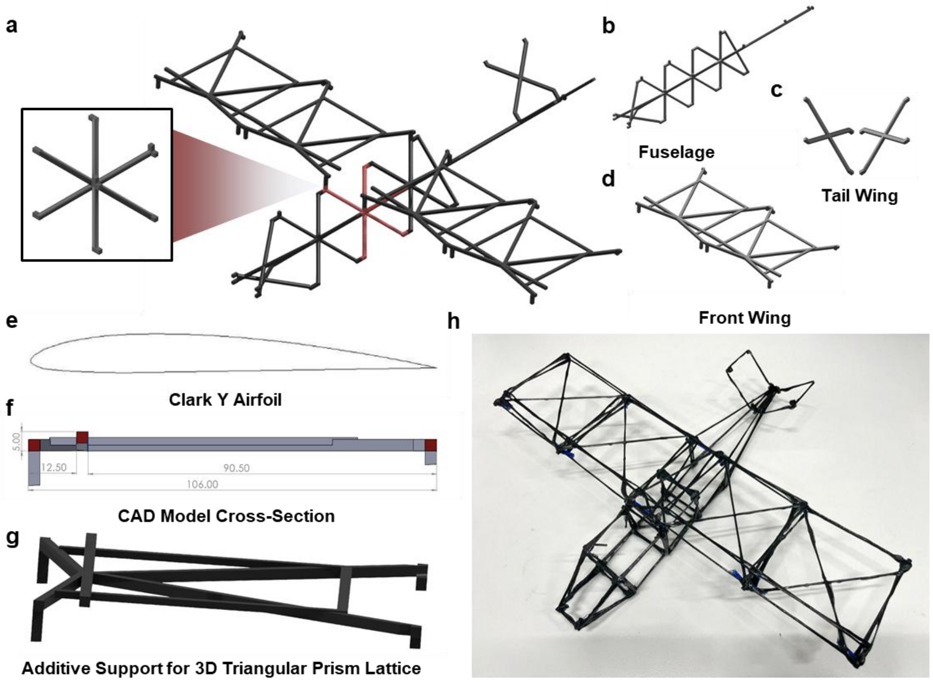

Supplementary Fig. 11. Miniature aircraft feasibility using multi-variant lattices.
a, Assembled 500 mm wingspan prototype. **b,** Fuselage composed of 5 lattice units to be connected by Type-B nodes. **c,** Tail wing constructed from two 2D square lattice units. **d,** Front wing assembled from four triangular-prism lattice cores. **e,** Clark-Y airfoil cross-section used for wing geometry**.** **f,** CAD model showing sectional layout. **g,** Fabricated lattice core derived directly from the airfoil profile. **h,** Final prototype assembly, highlighting the capacity of 3D node winding to realize aerodynamically relevant, fully continuous CFRP airframes.

Supplementary Tables

Supplementary Table 1. Lattice topologies considered for continuous carbon fiber winding.

Lattice	z	Min overlaps (per node)	Suitability
**SC**	6	3	✔ Benchmark, modular
**FCC**	12	6	✔ Isotropic, modular
**BCC**	8	8 (center node)	❌ Inefficient
**Triangular pyramid**	5–6	>6	❌ Limited stacking
**Hexagonal prism**	8–12	>6	❌ Non-rectangular

Supplementary Table 2. Types of CFRP unit cells fabricated.

Lattice	Horiz. Edges	Vert. Edges	Top/Bottom Diag.	Side Diag.	Loops	Sizes (mm)	Support
**SC**	✓	✓ (×2)	✗	✗	1, 2	25, 37, 50	Type A
**FCC**	✓	✓	✓	✓	1, 2	25, 37, 50	Type A
**SC**	✓	✓ (×2)	✗	✗	1, 2	25, 37, 50	Type B
**FCC**	✗	✗	✗	✓	1, 2	25, 37, 50	Type B

Supplementary Table 3. Continuity estimate across material classes.

Material Class	Main Discontinuities	Typical η_f	Effective η_{eff}	Notes
**SC (3D Winding)**	Start/end only; 3 overlaps/node	0.9–1.0	≈0.9–1.0	Benchmark case; minimal congestion, high fixity
**FCC (3D Winding)**	Start/end only; 6 overlaps/node	0.8–0.9	0.8–0.95	Isotropic; slightly reduced continuity but still high
**BCC (3D Winding)**	Central node with 8 overlaps	0.6–0.7	0.6–0.75	Congested nodes reduce fixity; prone to defects
**Laminate**	Z-ply breaks, XY edge cuts	XY: 0.85–0.98; Z: 0.1–0.5	0.3–0.7	Strong XY continuity; weak Z unless stitched
**Short-fiber**	Fiber ends in all directions	0.05–0.3 random; 0.2–0.6 aligned	0.1–0.5	Shear-lag governed; improves with aspect ratio and orientation.
**Particulate**	No fiber network	≈0	$E_m/E_c \sim$ 0.02–0.1	Only matrix continuity; particles act as local stiffeners

Supplementary Table 4. Cross-sectional properties of single CFRP columns.

Loops	Length (mm)	Fiber wt %	Area (mm²)	Eq. Diameter (mm)
1	50, 85, 150	22.4 ± 1.6	4.20	2.31 ± 0.537
2	50, 85, 150	26.7 ± 4.1	6.01	2.76 ± 0.611
5	50, 85, 150	36.3 ± 0.7	15.2	4.39 ± 0.503

Supplementary Table 5. Experimental compressive strengths of single CFRP columns compared with Euler buckling predictions.

Loop	L	λ	P_th (N)	P_exp (N)	Δ%
1	30	36.7	471.7	744	−36.6%
1	65	79.4	228.7	228.6	0.10%
1	130	159	82.8	82.8	−0.0%
2	30	29.6	1117	976.6	14.40%
2	65	64.1	837.4	836.8	0.10%
2	130	128	180.5	180.5	0.00%
5	30	24	1376	2198	−37.4%
5	65	52.1	1155	1155	0.10%
5	130	104	682.8	682.8	0.00%

Supplementary Table 6. Comparison of experimental compressive results for SC and FCC Type A lattices with imperfection-adjusted Euler predictions.

Lat.	Cell (mm)	ℓ	P_{ideal} (N)	φ(ℓ)	P_{th} (N)	P_{exp} (N)	Ratio
SC	25	1	1 755	0.35	614	314	0.51
SC	25	2	3 510	0.4	1 404	1 134	0.81
FCC	25	1	4 642	0.38	1 765	903	0.51
FCC	25	2	9 284	0.46	4 274	3 220	0.75
SC	37.5	1	784	0.35	274	474	1.73
SC	37.5	2	1 568	0.4	628	816	1.3
FCC	37.5	1	2 069	0.38	786	475	0.6
FCC	37.5	2	4 139	0.46	1 906	1 579	0.83
SC	50	1	439	0.35	154	132	0.86
SC	50	2	878	0.4	351	544	1.55
FCC	50	1	1 164	0.38	443	241	0.54
FCC	50	2	2 329	0.46	1 072	1 690	1.58

Supplementary Table 7. Summary of fracture modes and height recovery in SC and FCC Type B lattices.

Type	Loop	Size (mm)	Kink	Buckle	Delaminate	Recovery (%)
**SC**	1	25	✓	–	–	100
**SC**	1	37	✓	–	–	100
**SC**	1	50	–	✓	–	100
**SC**	2	25	✓	–	–	96
**SC**	2	37	✓	–	–	97.3
**SC**	2	50	–	✓	✓	100
**FCC**	1	25	✓	–	–	92
**FCC**	1	37	–	✓	–	100
**FCC**	1	50	–	✓	–	100
**FCC**	2	25	✓	–	–	96
**FCC**	2	37	–	✓	✓	94.6
**FCC**	2	50	–	✓	✓	100

Supplementary Table 8. Average slopes for SC, DC, and FCC beams

Lattice	Span (mm)	h (mm)	b (mm)	Loop	Slope (N/mm)
**SC**	100	22	30	1	45.82 ± 2.09
**SC**	100	30	22	1	21.40 ± 0.41
**SC**	140	22	30	1	33.69 ± 0.38
**SC**	140	30	22	1	11.16 ± 0.22
**SC**	180	22	30	1	20.27 ± 4.51
**SC**	180	30	22	1	5.81 ± 0.27
**DC**	100	22	30	1	24.41 ± 0.70
**DC**	100	30	22	1	69.40 ± 3.17
**DC**	140	22	30	1	8.33 ± 0.72
**DC**	140	30	22	1	15.44 ± 0.41
**DC**	180	22	30	1	5.43 ± 0.28
**DC**	180	30	22	1	13.06 ± 0.52
**DC**	100	22	30	2	184.45 ± 14.55
**DC**	100	30	22	2	402.81 ± 99.43
**DC**	140	22	30	2	44.23 ± 3.69
**DC**	140	30	22	2	77.04 ± 3.55
**DC**	180	22	30	2	26.71 ± 0.85
**DC**	180	30	22	2	66.31 ± 0.96
**FCC**	100	22	30	1	710.55 ± 106.08
**FCC**	100	30	22	1	674.04 ± 104.97
**FCC**	140	22	30	1	82.78 ± 5.78
**FCC**	140	30	22	1	97.87 ± 5.14
**FCC**	180	22	30	1	185.65 ± 25.57
**FCC**	180	30	22	1	226.26 ± 18.66
**FCC**	100	22	30	2	895.01 ± 38.13
**FCC**	100	30	22	2	765.48 ± 29.15
**FCC**	140	22	30	2	159.53 ± 33.87
**FCC**	140	30	22	2	188.69 ± 24.27
**FCC**	180	22	30	2	260.27 ± 16.82
**FCC**	180	30	22	2	401.89 ± 8.50

Supplementary Table 9. Top-5 optimized DC1 beam designs under 8 g mass constraint.

**Top 5 (mass ≤ 8.00 g)**								
**Rank**	*b* (= a_3) (mm)	*h* (mm)	a_1 (mm)	a_2 (mm)	a_4 (mm)	a_5 (mm)	mass (g)	k (N/mm)
**1**	20	34	50	65	65	50	7.99	6.33
**2**	16	36	63	54	54	63	8	6.11
**3**	24	30	78	35	35	78	8	5.74
**4**	24	29	70	43	43	70	7.91	5.36
**5**	20	31	73	42	42	73	7.9	5.31

Supplementary Table 10. System-level comparison of nylon, CFRP FCC lattice (CFRL1), and optimized DC-SC hybrid lattice (CFRL2) drone frames.

Parameter	Nylon	CFRL1	CFRL2	Change
**Frame mass**	100 g	60 g	21 g	−40% / −79%
**System mass**	504 g	457 g	420 g	−9.3% / −16.7%
**Avg. current**	9.70 A	7.93 A	7.32 A	−18 % / −25%
**Avg. power**	107.7 W	88.0 W	81.2 W	−18% / −25%
**Endurance**	12.25 min	15.0 min	16.25 min	+22 % / +33%
**Endurance gain**	–	+2.75 min	+4 min	–
