# Supplementary material for: Mesoscale carbon fiber lattices with foam-like weight and bulk strength
Source: Nat Commun. 2026 Apr 21;17:3615. doi: 10.1038/s41467-026-72105-4 (PMC13100039; doi:10.1038/s41467-026-72105-4)
Supplement: Supplementary file 2 — Description of Additional Supplementary File [file 41467_2026_72105_MOESM2_ESM.pdf]

The Description of Additional Supplementary Files

Supplementary Video 1. Real-time recordings of buckling tests on single CFRP columns, illustrating Euler instability and failure modes.

Supplementary Video 2. Compression tests of SC and FCC unit cells with synchronized force–displacement curves showing progressive lattice collapse.

Supplementary Video 3. Scanning electron microscope zoom-throughs highlighting kink bands and buckling fracture morphology in CFRP lattices.

Supplementary Video 4. Demonstration of drone frame comparison: weight measurement and hover endurance test contrasting nylon, CFRP, and lattice designs.

Supplementary Code 1. Python script implementing a genetic algorithm to compute shortest continuous f iber paths in SC and FCC lattices from 3D node coordinates, used for fabrication path planning.

Supplementary Code 2. Optimization code for lattice beam stiffness using experimental SC, DC, and FCC bending slopes, identifying lightweight (<8 g) configurations with target performance.

Supplementary Code 3. LibrePilot settings export including PID gains, stabilization modes, and flight control parameters used for quadcopter validation experiments.
