# Supplementary material for: Mesoscale carbon fiber lattices with foam-like weight and bulk strength
Source: Nat Commun. 2026 Apr 21;17:3615. doi: 10.1038/s41467-026-72105-4 (PMC13100039; doi:10.1038/s41467-026-72105-4)
Supplement: Supplementary file 11 — Transparent Peer Review file [file 41467_2026_72105_MOESM11_ESM.pdf]

nature portfolio

Peer Review File

Mesoscale Carbon Fiber Lattices with Foam-Like Weight and Bulk Strength

Corresponding Author: Professor Sung-Hoon Ahn

This manuscript has been previously reviewed at another journal that is not operating a transparent peer review scheme. The manuscript was considered suitable for publication without further review at Nature Communications.

This file contains all reviewer reports in order by version, followed by all author rebuttals in order by version.

Version 0:

Reviewer comments:

Reviewer #1

(Remarks to the Author)
The changes made to the text cover the current state of the art better than in the first submission. The reviewers' comments have been taken into account.

(Remarks on code availability)

Reviewer #2

(Remarks to the Author)
I would like to express my gratitude for the improved manuscript and inform the author that I have no further comments.

(Remarks on code availability)

Open Access This Peer Review File is licensed under a Creative Commons Attribution 4.0 International License, which permits use, sharing, adaptation, distribution and reproduction in any medium or format, as long as you give appropriate credit to the original author(s) and the source, provide a link to the Creative Commons license, and indicate if changes were made.
In cases where reviewers are anonymous, credit should be given to 'Anonymous Referee' and the source.
The images or other third party material in this Peer Review File are included in the article's Creative Commons license, unless indicated otherwise in a credit line to the material. If material is not included in the article's Creative Commons license and your intended use is not permitted by statutory regulation or exceeds the permitted use, you will need to obtain permission directly from the copyright holder.
To view a copy of this license, visit https://creativecommons.org/licenses/by/4.0/
